# Assessing heterogeneity of treatment effect analyses in health-related cluster randomized trials: A systematic review

**DOI:** 10.1371/journal.pone.0219894

**Published:** 2019-08-12

**Authors:** Monique Anderson Starks, Gillian D. Sanders, Remy Rene Coeytaux, Isaretta L. Riley, Larry R. Jackson, Amanda McBroom Brooks, Kevin L. Thomas, Kingshuk Roy Choudhury, Robert M. Califf, Adrian F. Hernandez

**Affiliations:** 1 Duke Clinical Research Institute, Duke University Medical Center, Durham, NC, United States of America; 2 Division of Cardiology, Department of Medicine, Duke University School of Medicine, Durham, NC, United States of America; 3 Department of Family and Community Medicine, Wake Forest School of Medicine; Winston-Salem, NC, United States of America; 4 Division of Pulmonary, Allergy, and Critical Care, Department of Medicine, Duke University School of Medicine, Durham, NC, United States of America; 5 Department of Biostatistics and Bioinformatics, Duke University, Durham, NC, United States of America; University of Zurich, SWITZERLAND

## Abstract

**Background:**

Cluster-randomized trials (CRTs) are being increasingly used to test a range of interventions, including medical interventions commonly used in clinical practice. Policies created by the NIH and the Food and Drug Administration (FDA) require the reporting of demographics and the examination of demographic heterogeneity of treatment effect (HTE) for individually randomized trials. Little is known about how frequent demographics are reported and HTE analyses are conducted in CRTs.

**Objectives:**

We sought to understand the prevalence of HTE analyses and the statistical methods used to conduct them in CRTs focused on treating cardiovascular disease, cancer, and chronic lower respiratory diseases. Additionally, we also report on the proportion of CRTs that reported on baseline demographics of its populations and conducted demographic HTE analyses.

**Data sources:**

We searched PubMed and Embase for CRTs published between 1/1/2010 and 3/29/2016 that focused on treating the top 3 Center for Disease Control causes of death (cardiovascular disease, chronic lower respiratory disease, and cancer).

Evidence Screening And Review: Of 1,682 unique titles, 117 abstracts were screened. After excluding 53 articles, we included 64 CRT publications and abstracted information on study characteristics and demographic information, statistical analysis, HTE analysis, and study quality.

**Results:**

Age and sex were reported in greater than 95.3% of CRTs, while race and ethnicity were reported in only 20.3% of CRTs. HTE analyses were conducted in 28.1% (n = 18) of included CRTs and 77.8% (n = 12) were prespecified analyses. Four CRTs conducted a demographic subgroup analysis. Only 6/18 CRTs used interaction testing to determine whether HTE existed.

**Conclusions:**

Baseline demographic reporting was high for age and sex in CRTs, but was uncommon for race and ethnicity. HTE analyses were uncommon and was rare for demographic subgroups, which limits the ability to examine the extent of benefits or risks for treatments tested with CRT designs.

## Introduction

Policies created by the NIH and the Food and Drug Administration (FDA) require the reporting of demographics and the examination of demographic heterogeneity of treatment effect (HTE). The stated objective is to evaluate possible differences in treatment effects across levels of baseline characteristics such as race, sex, or age for appropriate phase III randomized controlled trials [1–5]. The NIH inclusion policy requires demographic HTE analyses if any prior data strongly support evidence of possible HTE. Further, such phase III studies must be powered to detect differences in these subgroups by including adequate sample sizes[3]. The FDA’s Demographic Rule requires demographic HTE for all drug and biologic products and encourages HTE analyses for investigational devices [6]. In randomized trials where randomization is at the patient level, HTE is primarily assessed through interaction testing and is expressed in a statistical model as an interaction term between treatment group and baseline variable [7]. Pragmatic clinical trials (PCTs) are trials designed for the primary purpose of informing decision-makers regarding the comparative balance of benefits, burdens and risks of a biomedical or behavioral health intervention at the individual or population level. [8]. Cluster randomized trial (CRT) designs are commonly used in PCTs that are solicited and funded by the Patient-Centered Outcomes Research Institute (PCORI) and by the National Institutes of Health (NIH) [9]. These type of health-systems PCTs are expected to have larger patient populations and more women, elderly patients, and underrepresented minorities compared with individually randomized clinical trials. It is known that modeling cluster HTE is more complicated in CRTs compared with individually randomized trials given the difficulty with separating heterogeneity treatment effect from the cluster effect [10]. No standard requirements or approaches for demographic HTE testing in the context of cluster randomized PCTs have yet been established by either the NIH or the FDA.

In this study, we examine the prevalence of demographic reporting and HTE analyses in health-related PCTs using cluster designs. We focused on CRTs addressing cancer, cardiovascular disease, and chronic lower respiratory diseases (also referred to as “pulmonary”), the top 3 causes for mortality in the United States, as defined by the Centers for Disease Control and Prevention [11].

## Methods

### Overview

We performed a systematic review of health-related CRTs focused on strategies for treating cardiovascular disease, chronic lower respiratory pulmonary disease, and cancer published between January 1, 2010 and March 31, 2016. Due to the size of this body of literature, we focused our systematic review studies evaluating interventions for the three leading causes of death in the United States published in the aforementioned timeframe. A librarian conducted all searches using PubMed and Embase. To identify a CRT, a search strategy was employed that included “cluster randomized trial,” “pragmatic clinical trial,” “practical clinical trial,” or “group randomized trial” (see S1 Table for the full search strategy). International Classification of Diseases (ICD-10) codes were used to define the 3 therapeutic areas. For example, “chronic lower respiratory disease” comprised ICD-10 codes ranging from J40-J47 (bronchitis, chronic bronchitis, emphysema, chronic obstructive pulmonary disease, asthma, and bronchiectasis). ICD-10 codes for defining cardiovascular and cancer health systems CRTs for inclusion are listed in S1 Table. All health-related CRTs examined endpoints focused on individual-level morbidity and mortality.

### Study selection and data abstraction

An analysis plan was created before our investigation was initiated; we did not create or register a formal a priori protocol. Our specific key questions for the systematic review were: 1) How often and with what methods is HTE examined in published CRTs targeting the top 3 leading causes of death? 2) How often are HTE analyses conducted for demographic subgroups in published CRTs targeting the top 3 leading causes of death? and 3) How do these findings differ across clinical area, intervention type, and key characteristics of the population studied?

We developed specific inclusion and exclusion criteria (S2 Table) that were used by 4 investigators to independently review titles and abstracts for potential relevance to the key questions (2 reviewers per article).

#### Inclusion criteria

In general, we included CRTs in patients with cancer, cardiovascular disease, or chronic lower respiratory pulmonary disease conducted from 1/1/2010 and 3/31/2016. Only the primary main study result manuscript was included in our analysis. Only trials aimed at treating the three diseases were included; greater than or equal to 80% of the included population had to have the condition of interest.

#### Exclusion criteria

Studies that did not address the 3 conditions of interest were excluded. Additionally, CRTs that did not report patient-level outcomes were excluded. We also excluded prevention trials in which the population did not have the condition of interest (e.g. patients with risk factors [e.g. smoking, hyperlipidemia, diabetes) for the condition. Editorials, systematic reviews, meta-analyses, protocols, design manuscripts, and letters were excluded. Methods and subsequent manuscripts beyond primary or main study manuscript were also excluded.

Articles included by either reviewer underwent full-text screening. For the full-text stage, paired researchers (MAS, ILR, LRJII, RRC) independently reviewed the articles and made a decision for inclusion versus exclusion. If paired reviewers arrived at different decisions about inclusion versus exclusion or differed regarding the reason for exclusion, differences were reconciled through discussion and criteria review, or by a third investigator (GDS). Paired investigators with clinical and/or methodologic expertise abstracted data for each included article. One investigator extracted the data and the second reviewed the completed abstraction for accuracy and completeness. Disagreements were resolved by consensus or by involving a third investigator (GDS) if consensus could not be obtained.

### Data abstracted

Study characteristics included study identifiers (lead author, publication year, and ClinicalTrials.gov identifier), geographic location, funding source, study setting, intervention type, study enrollment numbers, number of clusters, number of patients, baseline characteristics of enrolled population, unit of analysis, and clinical area of interest. **Baseline characteristics** included age, sex, race and/or ethnicity, and socioeconomic status. For race and ethnicity, we assessed the number of studies that reported race and ethnicity according to federal Office of Management and Budget (OMB) standards [12]. Socioeconomic status (SES) included education, income, occupation, or insurance status. Abstracted **data on outcomes** included primary outcome, statistical test and category, inclusion of covariates for adjustment, reporting of study power and intraclass correlation, and subgroup analysis information (including information on statistical test and results). The primary outcome(s) was the outcome that evaluated the effect of the intervention and upon which the sample size and power calculations for the study are based. To assess the **quality of trials**, we used 5 design and analysis recommendations (cluster justification reported, ≥4 clusters per intervention group, allows for clustering in the sample size, uses matching or stratification, allows for clustering in the analysis) from a systematic review of primary care CRTs [13]. To assess the **quality of trial reporting**, we adopted recommendations from Eldridge et al. and CONSORT extension recommendations for CRTs [13,14]. These included cluster RCT identification in the title, reporting the estimate of intraclass correlation coefficient (ICC), number of clusters, baseline comparison of clusters and individuals, average cluster size, explanation of whether the primary analysis is conducted at the cluster versus individual patient- level, and reporting on loss of follow-up for clusters and individuals [13]. **Risk of bias** was assessed using a Cochrane Collaboration tool and included assessments of bias on random sequence generation, allocation concealment, blinding of participants and personnel, blinding of outcome assessment, incomplete outcome data, selective reporting, and other bias (see S7 Table for definitions and guidance for assessing risk of bias) [15].

### Analysis

Descriptive statistics of study characteristics, baseline characteristics, and outcomes overall and by clinical area (cardiovascular disease, cancer, and chronic lower respiratory disease) are presented. For trial conduct and reporting quality, we report the percentage of trials fulfilling each criterion overall and by clinical area. For risk of bias, we report on the number of trials with low risk, high risk, or unclear risk, overall and by clinical area.

## Results

### Literature search

**Fig 1:**
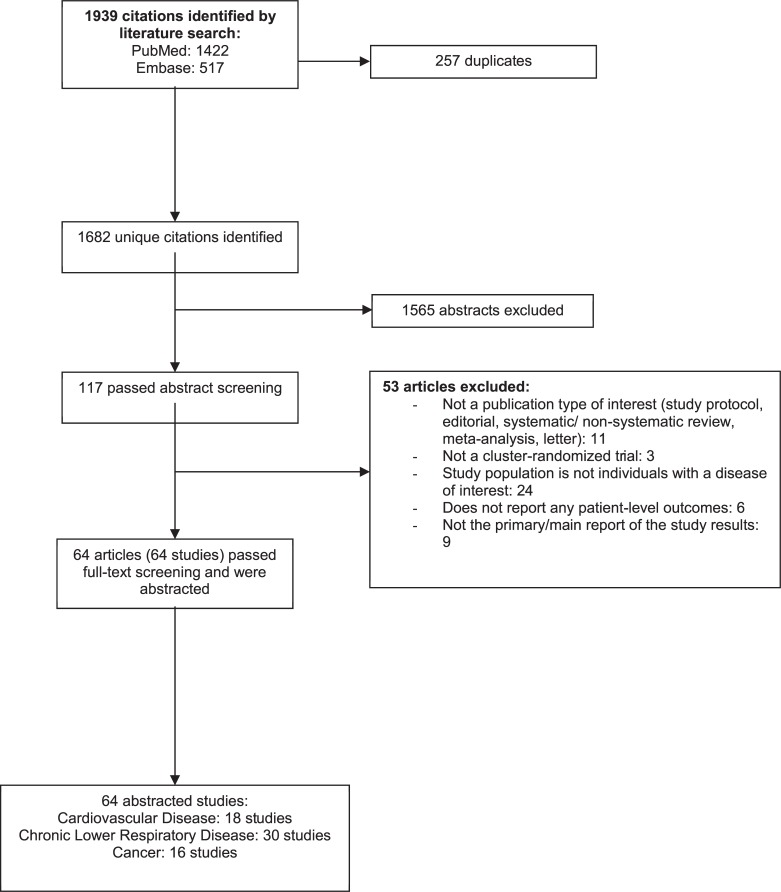
Flow diagram of the study selection process for the sample of 64 cluster-randomized trials included.

Fig 1 depicts the flow of articles through the literature search and screening process. Searches of PubMed and Embase yielded 1,682 unique citations.

After applying inclusion/exclusion criteria at the title-and-abstract level, 117 articles were retrieved and screened in full-text. Of these, 64 articles (64 studies) met eligibility criteria and proceeded to data abstraction. A detailed list of included articles is provided in S1 Text. S2 Text provides a complete list of articles excluded at the full-text screening stage (n = 53), with reasons for exclusion.

### Study and baseline characteristics

Of the 64 included studies, most were conducted in Europe (26/64), United States (15/64), and Australia/New Zealand (14/64) (Table 1).

**Table 1:** Study characteristics of included health systems cluster-randomized trials, overall and by disease area.

Trial characteristics	All (N = 64)	Cancer (N = 16)	Cardiovascular disease (N = 18)	Pulmonary disease (N = 30)
Median no. of patients enrolled (IQR)	468 (263.3–1003.5)	297.5 (243.5–505.5)	1405 (615.8–4307.5)	408 (294.0–652.0)
Median no. of clusters enrolled (IQR)	40 (19–98)	17 (12–87.8)	98 (39–174)	39 (27.3–53.8)
Geographic location, n (%)				
U.S.	15 (23.4)	2 (12.5)	7 (38.9)	6 (20.0)
Non-U.S.	49 (76.6)	14 (87.5)	11 (61.1)	24 (80.0)
Funding source, n (%)				
Government	20 (31.3)	5 (31.2)	5 (27.8)	10 (33.3)
Industry	2 (3.1)	0 (0.0)	1 (5.5)	1 (3.3)
Non-gov, non-industry	6 (9.4)	1 (6.25)	2 (11.1)	3 (10.0)
Mixed sources	21 (32.8)	6 (37.5)	5 (27.8)	10 (33.3)
Unclear	15 (23.4)	4 (25)	5 (27.8)	6 (20.0)
Setting, n (%)				
Clinic	40 (62.5)	10 (62.5)	6 (33.3)	24 (80.0)
Hospital	13 (20.3)	6 (37.5)	6 (33.3)	1 (3.3)
Emergency medical services	6 (9.4)	0 (0.0)	4 (22.2)	2 (6.6)
School/community	2 (3.1)	0 (0.0)	1 (5.6)	1 (3.3)
Other	3 (4.7)	0 (0.0)	1 (5.6)	2 (6.6)
Intervention, n (%)				
Devices	3 (4.7)	0 (0.0)	2 (11.1)	1 (3.3)
Drug or biologic	0 (0.0)	0 (0.0)	0 (0.0)	0 (0.0)
Quality improvement	34 (53.1)	11 (68.8)	9 (50.0)	14 (46.7)
Behavioral Interventions	12 (18.8)	2 (12.5)	4 (22.2)	6 (20.0)
Mixed Interventions	5 (7.8)	2 (12.5)	0 (0.0)	3 (10.0)
Other	10 (15.6)	1 (6.2)	3 (16.7)	6 (20.0)
Demographic characteristics				
Age, % reported, n (%)	61 (95.3)	16 (100)	18 (100)	27 (90.0)
Sex, % reported, n (%)	61 (95.3)	16 (100)	18 (100)	27 (90.0)
Race, % reported, n (%)	13 (20.3)	3 (18.8)	4 (22.2)	6 (20.0)
All NIH/OMB categories, n	1	1	0	0
White race only, n	3	0	2	1
One category, n	1	1	0	0
> 1 race category, n	7	1	2	4
Socioeconomic status, % reported (could be >1 per study), n (%)	30 (46.9)	9 (56.2)	5 (27.8)	16 (53.3)
Income level, n	4	0	2	2
Level of education, n	22	9	4	9
Employment status, n	7	0	2	5
Insurance status, n	5	1	1	3
Other, n	3	0	1	2

IQR, interquartile range

NIH/OMB minimum race and ethnicity categories: American Indian or Alaska Native, Asian, Black or African American, Hispanic or Latino, Native Hawaiian or Other Pacific Islander, White

Most included CRTs were conducted in a clinic (62.5%) or hospital setting (20.3%) and tested quality improvement (53.1%) or behavioral (18.8%) interventions. The median number of patients enrolled was 468 (IQR: 243.3–1003.5) with a median number of clusters of 40 (IQR: 19–98). Cardiovascular CRTs included the largest median number of patients compared with pulmonary or cancer CRTs, respectively. Common funding sources were either government (31.3%) or multiple sources (32.8%).

Most of the CRTs reported the age (95.3%) and sex (95.3%) of included patients (Table 1, S3 Table). Cancer and cardiovascular CRTs had 100% reporting of age and sex vs 90% reporting for pulmonary CRTs. Reporting of race and ethnicity of included health systems CRTs was uncommon, both overall (20.3%) and by disease area (cancer: 18.8%; cardiovascular: 22.2%; pulmonary: 20.0%). When race and ethnicity were reported, only 1 CRT listed NIH/OMB race and ethnicity categories. Race/ethnicity reporting was 73% for CRTs conducted in the United States, and only 4.1% for CRTs conducted in other countries. An SES measure was reported for 46.9% of CRTs. When reporting SES, level of education was the most commonly reported SES measure (22/30 [73.3%]) (S3 Table).

### Primary outcomes, statistical design, and results

Of 64 included CRTs, 93.8% reported clearly specified the primary outcome(s); 16 studies reported more than one primary outcome (Table 2). Of studies reporting a primary outcome, most CRTs examined a patient-reported outcome (46.7%). Clinical, process of care, and survival/mortality outcomes were reported in 25.0%, 16.7%, and 13.3% of included CRTs (Table 2).

**Table 2:** Primary outcome and statistical analysis for health system cluster-randomized trial cohort.

	All (N = 64)	Cancer (N = 16)	Cardiovascular disease (N = 18)	Pulmonary disease (N = 30)
**Primary outcome (s) reported, n (%)**^**&**^	**60 (93.8)**	**15 (93.8)**	**18 (100)**	**27 (90.0)**
Patient-reported outcome (PRO), n	28 (46.7)	10 (66.7)	3 (16.7)	15 (55.6)
Clinical outcome, n	15 (25.0)	3 (20.0)	5 (27.8)	7 (25.9)
Process outcome, n	10 (16.7)	2 (13.3)	6 (33.3)	2 (7.4)
Economic outcome, n	1 (1.7)	0	0	1 (3.3)
Behavioral outcome, n	1 (1.7)	0	0	1 (3.3)
Mortality/survival, n	8 (13.3)	0	7 (38.9)	1 (3.3)
**Statistical test, n (%)**^**&**^	**61 (95.3)**	**14 (87.5)**	**17 (94.4)**	**30 (100)**
*Tests Accounting for Clustering*, *n*	*50 (81*.*9)*	*13 (92*.*9)*	*14 (82*.*4)*	*23 (76*.*7)*
Mixed models	29 (58.0)	7 (53.8)	5 (35.7)	17 (73.9)
Generalized estimating equations	13 (26.0)	6 (46.2)	4 (28.6)	3 (13.0)
Cox model with clustering	1 (2.0)	0	1 (7.1)	0
Other models with clustering-CACE, differences 2 time points, ANOVA/ANCOVA, proportional odds model	7 (14.0)	0	5 (35.7)	2 (8.7)
*Tests not Accounting for Clustering*, *n*	*12 (19*.*6)*	*2 (14*.*3)*	*3 (17*.*6)*	*7 (23*.*3)*
Comparison of means	5 (41.7)	0	2 (66.7)	3 (42.9)
Generalized linear model	5 (41.7)	1 (50.0)	1 (33.3)	3 (42.9)
Logistic regression, no clustering	1 (8.3)	0	0	1 (14.3)
Cox model, no clustering	1 (8.3)	1 (50.0)	0	0
**Treatment effect present (primary outcome), n (%)**^**^**^	**24 (37.5)**	**5 (31.3)**	**5 (27.8)**	**14 (46.7)**

^&^ Some studies reported more than one primary outcome and more than one statistical test.

*Mixed models include generalized linear mixed model, mixed effect model, multilevel linear regression, linear mixed model, multilevel linear model, logistic regression with clustering, and binomial regression with clustering.

^†^Demographic variables include primarily individual level demographics- age, sex, race, ethnicity, socioeconomic status (education, income, occupation), or insurance status. Most models included >1 individual-level demographic covariate.

^Treatment effect present refers to statistical significance.

ANOVA, analysis of variance; ANCOVA, analysis of covariance; CACE, complier average causal effectDetails of the statistical approach to evaluating differences in study arms were included in 95.3% (n = 61) of our sample. Of those, 67.3% used statistical methods that accounted for clustering by utilizing mixed models (n = 29 [58.0%]) and regression with generalized estimating equations (n = 13 [26.0%]). An estimated 92.9% of cancer CRTs, 82.4% of cardiovascular CRTs, and 76.7% of pulmonary CRTs utilized statistical methods accounting for clustering. Approximately 19.6% of CRTs did not account for clustering. These methods included simple comparison of means (n = 5), generalized linear models (n = 5), regression models without accounting for clustering (n = 1), and simple Cox models (n = 1). Other details of the statistical design are listed in S4 Table.

### Heterogeneity of treatment effect

Of all included CRTs, only 1 trial presented the power analysis for a subgroup analysis [16]. Only 28.1% of the CRTs performed subgroup analysis. CRTs focused on cardiovascular disease (50%) were more likely to report HTE analyses, compared with pulmonary CRTs (26.7%) and cancer CRTs (6.3%) (Table 3).

**Table 3:** Statistical information and results for heterogeneity of treatment effect analyses for included health systems cluster-randomized trials.

	All (N = 64)	Cancer (N = 16)	Cardiovascular disease (N = 18)	Pulmonary disease (N = 30)
**Power analysis for subgroup, n (%)**	**1 (1.5)**	**0 (0.0)**	**0 (0.0)**	**1 (3.3)**
**Type of subgroup analyses**				
Any subgroup analysis performed, n (%)	18 (28.1)	1 (6.2)	9 (50.0)	8 (26.7)
Demographic subgroup analysis	4 (22.2)	0	3 (33.3)	1 (12.5)
Prespecified Subgroup Analyses	12 (66.7)	1 (100)	7 (77.8)	4 (50.0)
**Subgroup statistical Test**	**18 (28.1)**	**1 (6.2)**	**9 (50.0)**	**8 (26.6)**
Same as primary outcome (within-group comparison)	5 (27.5)	0	2 (22.2)	3 (37.5)
Interaction testing	8 (44.4)	1 (100)	5 (55.6)	2 (25.0)
Not reported	5 (27.5)	0	2 (22.2)	3 (37.5)
HTE found for any subgroup, n (%)^*^	**8 (44.4)**	**0 (0.0)**	**5 (55.6)**	**3 (37.5)**
HTE found for demographic subgroup, n (%)^*^	**1 (25.0)**	**0**	**0**	**1 (100%)**

*Statistical significance found for subgroup analysis.

Demographic subgroup analyses were uncommon among included CRTs (4/64 or 6.3%). No cancer, three cardiovascular [17–19], and one pulmonary [16,20] CRTs performed demographic HTE analyses.

Of 18 trials reporting HTE analyses, 12 (66.7%) of the CRTs described its subgroup analyses as *a priori*, prespecified, or planned. The type of statistical test for the subgroup analysis was reported for 13 (77.8%) CRTs. Five of the 13 CRTs reported using separate tests for treatment effects within each of the levels of the baseline characteristic under evaluation, while 8 of the 13 reported using a statistical test for interaction. Eight studies found significant heterogeneity of treatment effect for study arms: 0 for cancer, 5 for cardiovascular [18,19,21,22], and 3 for pulmonary CRTs [20,23,24]) (Table 3). Of these, only 1 study demonstrated HTE by a demographic subgroup [20].

### Design and reporting quality

Quality varied for the design of CRTs examined in our study. Only 37.5% of CRTs reported a justification for a cluster design, 57.8% used techniques to ensure balance at the cluster level, and 60% accounted for clustering in the sample size calculations. A majority allowed for clustering in the statistical analysis and 89% of CRTs had at least 4 clusters per arm. Only 7.8% of trials had 100% compliance for quality assessment and reporting (S5 Table) [20, 25–28]. Information on risk of bias is reported in the appendix (S6 Table).

## Discussion

Cluster randomized trials are increasing being employed to evaluate diagnostics and therapeutic strategies in medicine. When randomization is at the individual patient level, both NIH and FDA have requirements specifying the reporting of demographics and the examination of certain types of treatment heterogeneity. For cluster randomized trials, no similar requirements have yet been published or little is known about how often these trials currently report and examine HTE. In this systematic review of 64 cluster randomized PCs (2010–2016), we found that the reporting of baseline demographics was low for race and ethnicity and socioeconomic status. Heterogeneity of treatment effect analyses were uncommon and demographic HTE was rare. When subgroup analyses were conducted, more often than not, interaction testing was not used. Finally, overall quality assessment and reporting quality for included CRTs were low.

The *Consolidated Standards of Reporting Trials (CONSORT) statement*: *Extension to Cluster Randomized Trials*, developed to improve the reporting of CRTs, recommends the routine reporting of baseline and demographic characteristics for cluster and individual research participants, given the inherent higher risk of chance bias in CRTs [14]. There was a high prevalence of age and sex reporting in included CRTs; however, race and ethnicity were reported for only 20.3% of CRTs (Table 2). When assessed by country, U.S. trials reported race and ethnicity in 73% of CRTs, while non-U.S. trials reported race and ethnicity in only 4.1% of CRTs. In general, NIH-funded studies and individually randomized RCTs with FDA oversight are required to report the race and ethnicity of trial participants; however, such policies may not exist for CRTs conducted in other countries [3,6].

We found that HTE analyses in health-related CRTs are uncommon. When conducted, it was rare to provide information for power analysis. For most studies reporting HTE analyses, separate tests of treatment effects within each of the levels of the baseline variable were performed. Only 8 CRTs reported using interaction testing, which is deemed appropriate for subgroup analysis [19–22,25,29–31]. Subgroup HTE analyses examined baseline characteristics (age, sex, and SES) in a small number of studies, but no subgroup analyses were conducted for race and ethnicity. To our knowledge, this is the first publication to report on the quality and nature of subgroup analyses in health systems CRTs. Given that the NIH and FDA have policies for demographic reporting and subgroup analyses [2,6], investigators seeking federal funding for CRTs could benefit from formal guidance on demographic reporting, trial and statistical design considerations, and optimal techniques and expectations for demographic subgroups.

### Limitations

We note a number of limitations to our analysis. First, because most of the studies we evaluated were conducted outside of the United States, it is unclear whether HTE analyses are encouraged or required by funders and/or agencies with oversight. While these non-U.S. trials do not fall under reporting requirements mandated by the FDA and NIH, international CONSORT guidelines for CRTs recommend baseline demographic reporting by intervention group. Second, although an attempt was made to identify all CRTs aimed at treating cardiovascular disease, lower respiratory disease, or cancer published during this contemporary time period, we may have missed some articles. Third, some salient trial aspects deemed to be missing from CRT publications may have been present in study protocols (e.g., power analyses) or subsequent publications (e.g., other subgroup analyses). However, the scope of this manuscript was to understand the degree to which this information is reported in primary manuscripts, which is commonly standard to primary publications for individually randomized clinical trials published in higher-tier journals. Interpretation of components of risk of bias could have influenced coding. Fourth, if identification of bias assessment was not obvious, it was marked as “unclear.” Fifth, because we chose the top 3 causes of mortality in the U.S. as the clinical areas of focus for this analysis, our results may not be generalizable to other medical conditions. Finally, our review was primarily a methodology review. Although our reporting period is through March 2016, we still believe the findings are relevant to this methodological topic of reporting and HTE analyses.

### Conclusions

Cluster-randomized trials are being increasingly used to compare commonly used medical interventions. Expectations and guidance should be established for baseline demographic reporting and HTE analysis in CRTs.

## Supporting information

S1 Checklist PRISMA 2009 checklist. (DOC)

S1 Table Exact search strings. (DOCX)

S2 Table Screening inclusion/exclusion criteria. (DOCX)

S3 Table Characteristics of included studies. (DOC)

S4 Table ICC and power estimate for income studies. (DOCX)

S5 Table Design and reporting quality for included health systems CRTs. (DOCX)

S6 Table Risk of bias for included CRTS. (DOCX)

S7 Table The cochrane collaboration’s tool for assessing risk of bias. (DOCX)

S1 Text List of included studies. (DOCX)

S2 Text List of excluded studies. (DOCX)

S1 Dataset Raw extraction data of included studies. (XLSX)

S2 Dataset Review of subgroups for prespecified status. (XLSX)
