# Supplementary material for: Assessing heterogeneity of treatment effect analyses in health-related cluster randomized trials: A systematic review
Source: PLoS One. 2019 Aug 12;14(8):e0219894. doi: 10.1371/journal.pone.0219894 (PMC6690528; doi:10.1371/journal.pone.0219894)
Supplement: S1 Table — (DOCX) [file pone.0219894.s002.docx]

**S1 Appendix. Exact Search Strings**

For each database, we report below: (1) individually-designed search strategies for each of the three clinical areas considered and (2) the total number of unique citations returned when all three searches were combined in an OR string.

**PubMed^®^ search strategy (March 29, 2016)**

Cardiovascular Disease:

|  | Terms | Results |
| --- | --- | --- |
| **#1** | ("Pragmatic Clinical Trial" [Publication Type] OR (("Clinical Trial" [Publication Type] OR trial[tiab] OR trials[tiab]) AND (Cluster*[tiab] OR "Cluster Analysis"[Mesh] OR pragmatic[tiab]) AND random*[tiab]) OR pragmatic trial*[tiab] OR pragmatic clinical trial*[tiab] OR practical trial*[tiab] OR practical clinical trial*[tiab] OR group randomized[tiab] OR group randomization[tiab] OR group randomised[tiab] OR group randomisation[tiab]) NOT (Editorial[ptyp] OR Letter[ptyp] OR Case Reports[ptyp] OR Comment[ptyp]) NOT ("Review" [Publication Type] OR "Meta-Analysis" [Publication Type]) NOT (animals[mh] NOT humans[mh]) AND English[la] AND ("2010"[Date - Publication] : "3000"[Date - Publication]) | 6944 |
| **#2** | “Cardiovascular Diseases”[MeSH] OR “Cardiology”[MeSH] OR "Atrial Fibrillation"[MeSH] OR "heart disease"[tiab] OR "atrial fibrillation"[tiab] OR “hypertensive heart disease”[tiab] OR “rheumatic heart disease”[tiab] OR “cardiomyopathy”[tiab] OR “endocarditis”[tiab] OR “coronary artery disease*”[tiab] OR “coronary arterial disease*”[tiab] OR "heart arrest"[tiab] OR "cardiac arrest"[tiab] OR "cardiac death"[tiab] OR "sudden death" OR "valvular heart disease*"[tiab] OR arrhythmia*[tiab] OR "heart failure"[tiab] OR "pulmonary hypertension"[tiab] OR pericarditis[tiab] OR myocarditis[tiab] OR tamponade[tiab] OR angina[tiab] OR nstemi[tiab] OR stemi[tiab] OR "coronary artery disease"[tiab] OR atherosclerosis[tiab] OR "pulmonary embolism"[tiab] OR "pericardial effusion"[tiab] OR "ventricular tachycardia"[tiab] OR "ventricular fibrillation"[tiab] OR " sick sinus syndrome"[tiab] OR preexcitation[tiab] OR "pulmonary heart disease"[tiab] OR "aortic stenosis"[tiab] OR "aortic insufficiency"[tiab] OR "mitral stenosis"[tiab] OR "mitral regurgitation"[tiab] OR "tricuspid stenosis"[tiab] OR "tricuspid insufficiency"[tiab] OR "pulmonic stenosis"[tiab] OR "pulmonic insufficiency"[tiab] OR "myocardial infarction"[tiab] OR "ischemic heart disease"[tiab] OR "acute coronary syndrome"[tiab] | 2208905 |
| **#3** | #1 AND #2 | 634 |

Cancer:

|  | Terms | Results | | |
| --- | --- | --- | --- | --- |
| **#1** | ("Pragmatic Clinical Trial" [Publication Type] OR (("Clinical Trial" [Publication Type] OR trial[tiab] OR trials[tiab]) AND (Cluster*[tiab] OR "Cluster Analysis"[Mesh] OR pragmatic[tiab]) AND random*[tiab]) OR pragmatic trial*[tiab] OR pragmatic clinical trial*[tiab] OR practical trial*[tiab] OR practical clinical trial*[tiab] OR group randomized[tiab] OR group randomization[tiab] OR group randomised[tiab] OR group randomisation[tiab]) NOT (Editorial[ptyp] OR Letter[ptyp] OR Case Reports[ptyp] OR Comment[ptyp]) NOT ("Review" [Publication Type] OR "Meta-Analysis" [Publication Type]) NOT (animals[mh] NOT humans[mh]) AND English[la] AND ("2010"[Date - Publication] : "3000"[Date - Publication]) | 6944 | | |
| **#2** | “Neoplasms”[MeSH] OR cancer[tiab] OR cancers[tiab] OR cancerous[tiab] OR neoplasm[tiab] OR neoplasms[tiab] OR oncology[tiab] OR oncologic[tiab] OR carcinoma[tiab] OR carcinomas[tiab] OR lymphoma[tiab] OR leukemia[tiab] OR "mycosis fungiodes"[tiab] OR "sezary disease"[tiab] OR "multiple myeloma"[tiab] OR plasmacytoma[tiab] OR sarcoma[tiab] OR macroglobulinemia[tiab] OR "lymphoproliferative disease"[tiab] | 3223267 | | |
| **#3** | #1 AND #2 | 464 | | |
| Chronic Lower Respiratory Disease: | | | | |
|  | Terms | | Results |  |
| **#1** | ("Pragmatic Clinical Trial" [Publication Type] OR (("Clinical Trial" [Publication Type] OR trial[tiab] OR trials[tiab]) AND (Cluster*[tiab] OR "Cluster Analysis"[Mesh] OR pragmatic[tiab]) AND random*[tiab]) OR pragmatic trial*[tiab] OR pragmatic clinical trial*[tiab] OR practical trial*[tiab] OR practical clinical trial*[tiab] OR group randomized[tiab] OR group randomization[tiab] OR group randomised[tiab] OR group randomisation[tiab]) NOT (Editorial[ptyp] OR Letter[ptyp] OR Case Reports[ptyp] OR Comment[ptyp]) NOT ("Review" [Publication Type] OR "Meta-Analysis" [Publication Type]) NOT (animals[mh] NOT humans[mh]) AND English[la] AND ("2010"[Date - Publication] : "3000"[Date - Publication]) | | 6944 |  |
| **#2** | “Respiratory Tract Diseases”[MeSH] OR "Pulmonary Disease, Chronic Obstructive"[MeSH] OR "COPD"[tiab] OR "chronic obstructive pulmonary disease"[tiab] OR "bronchitis"[tiab] OR “chronic lung disease”[tiab] OR “obstructive airway disease”[tiab] OR “Emphysema”[tiab] OR “asthma”[tiab] OR “bronchial disease”[tiab] OR “bronchiectasis”[tiab] | | 1193792 |  |
| **#3** | #1 AND #2 | | 395 |  |

All PubMed searches combined: 1422 unique citations

**Embase search strategy (March 29, 2016)**

Cardiovascular Disease:

|  | Terms | Results |
| --- | --- | --- |
| **#1** | ((('clinical trial'/exp OR trial:ab,ti OR trials:ab,ti) AND ((cluster*:ab,ti OR 'cluster analysis'/exp OR pragmatic:ab,ti) AND random*:ab,ti)) OR "pragmatic trial*":ab,ti OR "pragmatic clinical trial*":ab,ti OR "practical trial*":ab,ti OR "practical clinical trial*":ab,ti OR "group randomized":ab,ti OR "group randomization":ab,ti OR "group randomised":ab,ti OR "group randomization":ab,ti) NOT ('case report'/exp OR 'case study'/exp OR 'editorial'/exp OR 'letter'/exp OR 'note'/exp OR 'systematic review'/exp OR 'meta analysis'/exp OR [conference abstract]/lim) AND [humans]/lim AND [english]/lim AND [2010-2016]/py AND [embase]/lim NOT [medline]/lim | 1888 |
| **#2** | 'cardiovascular disease'/exp OR 'cardiology'/exp OR 'atrial fibrillation'/exp OR "heart disease":ab,ti OR "atrial fibrillation":ab,ti OR “hypertensive heart disease”:ab,ti OR “rheumatic heart disease”:ab,ti OR “cardiomyopathy”:ab,ti OR “endocarditis”:ab,ti OR “coronary artery disease*”:ab,ti OR “coronary arterial disease*”:ab,ti OR "heart arrest":ab,ti OR "cardiac arrest":ab,ti OR "cardiac death":ab,ti OR "sudden death" OR "valvular heart disease*":ab,ti OR arrhythmia*:ab,ti OR "heart failure":ab,ti OR "pulmonary hypertension":ab,ti OR pericarditis:ab,ti OR myocarditis:ab,ti OR tamponade:ab,ti OR angina:ab,ti OR nstemi:ab,ti OR stemi:ab,ti OR "coronary artery disease":ab,ti OR atherosclerosis:ab,ti OR "pulmonary embolism":ab,ti OR "pericardial effusion":ab,ti OR "ventricular tachycardia":ab,ti OR "ventricular fibrillation":ab,ti OR " sick sinus syndrome":ab,ti OR preexcitation:ab,ti OR "pulmonary heart disease":ab,ti OR "aortic stenosis":ab,ti OR "aortic insufficiency":ab,ti OR "mitral stenosis":ab,ti OR "mitral regurgitation":ab,ti OR "tricuspid stenosis":ab,ti OR "tricuspid insufficiency":ab,ti OR "pulmonic stenosis":ab,ti OR "pulmonic insufficiency":ab,ti OR "myocardial infarction":ab,ti OR "ischemic heart disease":ab,ti OR "acute coronary syndrome":ab,ti | 3686974 |
| **#3** | #1 AND #2 | 252 |

Cancer:

|  | Terms | Results | | |
| --- | --- | --- | --- | --- |
| **#1** | ((('clinical trial'/exp OR trial:ab,ti OR trials:ab,ti) AND ((cluster*:ab,ti OR 'cluster analysis'/exp OR pragmatic:ab,ti) AND random*:ab,ti)) OR "pragmatic trial*":ab,ti OR "pragmatic clinical trial*":ab,ti OR "practical trial*":ab,ti OR "practical clinical trial*":ab,ti OR "group randomized":ab,ti OR "group randomization":ab,ti OR "group randomised":ab,ti OR "group randomization":ab,ti) NOT ('case report'/exp OR 'case study'/exp OR 'editorial'/exp OR 'letter'/exp OR 'note'/exp OR 'systematic review'/exp OR 'meta analysis'/exp OR [conference abstract]/lim) AND [humans]/lim AND [english]/lim AND [2010-2016]/py AND [embase]/lim NOT [medline]/lim | 1888 | | |
| **#2** | 'neoplasm'/exp OR cancer:ab,ti OR cancers:ab,ti OR cancerous:ab,ti OR neoplasm:ab,ti OR neoplasms:ab,ti OR oncology:ab,ti OR oncologic:ab,ti OR carcinoma:ab,ti OR carcinomas:ab,ti OR lymphoma:ab,ti OR leukemia:ab,ti OR "mycosis fungiodes":ab,ti OR "sezary disease":ab,ti OR "multiple myeloma":ab,ti OR plasmacytoma:ab,ti OR sarcoma:ab,ti OR macroglobulinemia:ab,ti OR "lymphoproliferative disease":ab,ti | 4312330 | | |
| **#3** | #1 AND #2 | 143 | | |
| Chronic Lower Respiratory Disease: | | | | |
|  | Terms | | Results |  |
| **#1** | ((('clinical trial'/exp OR trial:ab,ti OR trials:ab,ti) AND ((cluster*:ab,ti OR 'cluster analysis'/exp OR pragmatic:ab,ti) AND random*:ab,ti)) OR "pragmatic trial*":ab,ti OR "pragmatic clinical trial*":ab,ti OR "practical trial*":ab,ti OR "practical clinical trial*":ab,ti OR "group randomized":ab,ti OR "group randomization":ab,ti OR "group randomised":ab,ti OR "group randomization":ab,ti) NOT ('case report'/exp OR 'case study'/exp OR 'editorial'/exp OR 'letter'/exp OR 'note'/exp OR 'systematic review'/exp OR 'meta analysis'/exp OR [conference abstract]/lim) AND [humans]/lim AND [english]/lim AND [2010-2016]/py AND [embase]/lim NOT [medline]/lim | | 1888 |  |
| **#2** | 'respiratory tract disease'/exp OR "COPD":ab,ti OR "chronic obstructive pulmonary disease":ab,ti OR "chronic obstructive lung disease":ab,ti OR "bronchitis":ab,ti OR “chronic lung disease”:ab,ti OR “obstructive airway disease”:ab,ti OR “Emphysema”:ab,ti OR “asthma”:ab,ti OR “bronchial disease”:ab,ti OR “bronchiectasis”:ab,ti | | 2181676 |  |
| **#3** | #1 AND #2 | | 188 |  |

All Embase searches
