# Supplementary material for: Assessing heterogeneity of treatment effect analyses in health-related cluster randomized trials: A systematic review
Source: PLoS One. 2019 Aug 12;14(8):e0219894. doi: 10.1371/journal.pone.0219894 (PMC6690528; doi:10.1371/journal.pone.0219894)
Supplement: S2 Table — (DOCX) [file pone.0219894.s003.docx]

S2 Table. Screening Inclusion/Exclusion Criteria

| **Element** | **Inclusion Criteria** | **Exclusion Criteria** |
| --- | --- | --- |
| **Study Design** | • Cluster-randomized trials | • All study designs other than cluster-randomized trials |
| **Clinical Areas of Interest** | **Patients must have a condition in one of the clinical areas of interest listed below:**    **HEART DISEASE:** • All forms of rheumatic fever or heart disease  o Rheumatic pericarditis, endocarditis, myocarditis, acute rheumatic heart disease, rheumatic mitral disease, rheumatic aortic disease, rheumatic tricuspid disease, combined valve disease, myocarditis  • Hypertensive heart disease with or without heart failure • Angina  o Unstable angina, angina pectoris  • ST elevation myocardial infarction (STEMI)  • Non-ST elevation myocardial infarction (NSTEMI)  • Atrial septal defect (ASD), ventricular septal defect (VSD), rupture, and other acute myocardial infarction (AMI) complications  o Hemopericardium, postinfarction angina, thrombosis of atrium, rupture of cardiac wall, ruptured chordae tendineae, ruptured papillary muscle  • Acute coronary thrombosis, Dressler’s syndrome, other forms of ischemic heart disease • All forms of atherosclerosis, aneurysm, coronary artery dissection • Pulmonary embolism • Pulmonary hypertension, pulmonary heart disease  o Includes cor pulmonale and pulmonary heart disease  • Diseases of pulmonary vessels  o Arteriovenous fistula, aneurysm, other diseases  • Pericarditis • Pericardial effusion, other pericarditis, or tamponade  • All valve disease  o Mitral valve insufficiency, mitral valve prolapse, mitral valve stenosis, mitral valve disorders, aortic valve insufficiency, aortic valve stenosis, tricuspid stenosis, tricuspid valve insufficiency, pulmonary stenosis, pulmonary insufficiency  • Endocarditis • Myocarditis • Cardiomyopathy • Atrioventricular (AV) block, fascicular block, bundle branch block • Long QT, other block, preexcitation • Cardiac arrest • Ventricular tachycardia • Atrial fibrillation/flutter • Ventricular fibrillation, flutter, premature depolarization, sick sinus syndrome (SSS) • Heart failure    **CANCER (All neoplasms except where listed)** • Cancer of the lip • Head and neck • GI tract - stomach, small intestine, large intestine, rectum, anus • Liver • Gallbladder • Biliary tract • Pancreas • Spleen and other digestive system • Nasal cavity and inner ear • Sinuses - maxillary, ethmoid, frontal, sphenoid, overlapping • Glottis or larynx • Trachea • Bronchus, lung • Thymus • Heart, mediastinum • Upper and lower respiratory tract • Bones of limb • Other bones of face, vertebrae, and clavicle • Skin • Pleura, peritoneum, pericardium • Kaposi's sarcoma, any organ • Peripheral nerves • Peritoneum • Connective and soft • Breast • Reproductive organs - female • Reproductive organs - male • Urogenital system  • Renal • Eye • Meninges • Brain, spinal cord, cranial nerves • Thyroid • Adrenal gland • Glands, endocrine • Head, face and neck, limbs, abdomen, pelvis • Lymph nodes • Secondary malignant neoplasm of other organs • Secondary malignant neoplasm • Transplanted organ • Hodgkin lymphoma • Follicular lymphoma • Small B-cell lymphoma • Other lymphoma, mycosis fungoides, Sézary disease • Unspecified B-cell lymphoma • T-cell lymphoma • Waldenstrom macroglobulinemia, heavy chain disease, malignant immunoproliferative disease • Multiple myeloma, plasma cell leukemia, solitary plasmacytoma • Leukemia • Acute myeloblastic leukemia, acute promyelocytic leukemia, other leukemia • Chronic myleomonocytic leukemia • Acute erythroid, mast cell leukemia • Chronic leukemia, acute leukemia • Histiocytosis, malignant mast cell tumor    **CHRONIC LOWER RESPIRATORY DISEASES:** • Bronchitis • Chronic bronchitis • Emphysema • COPD • Asthma • Bronchiectasis | • Any conditions not specified at left, e.g., stroke, influenza, pneumonia.    • Less than 80% of the study population has a condition of interest specified at left    • Prevention trials in which the population does not have any of the conditions specified at left.  (Note: presence of diabetes, hyperlipidemia, smoking history, etc are not sufficient.) |
| **Outcomes** | Reports at least one patient-level outcome | Does not report any patient-level outcomes |
| **Publication Type** | • English-language only • Published on or after January 1, 2010  • Primary/main study result articles | • Editorials, systematic or non-systematic reviews and meta-analyses, protocols, letters, abstract-only publications, conference or poster abstracts, withdrawn or retracted publications • Secondary analysis articles |
