# Supplementary material for: Assessing heterogeneity of treatment effect analyses in health-related cluster randomized trials: A systematic review
Source: PLoS One. 2019 Aug 12;14(8):e0219894. doi: 10.1371/journal.pone.0219894 (PMC6690528; doi:10.1371/journal.pone.0219894)
Supplement: S3 Table — (DOC) [file pone.0219894.s004.doc]

**S3 Table. Characteristics of Included Studies**

**Appendix Table E: Characteristics of Included CRTs**

| **Author, Year**  **Journal**  **ACRONYM** | **Disease Area of Interest** | **Study Details** | **Patient Characteristics** | **Outcomes** |
| --- | --- | --- | --- | --- |
| Abernathy, 2013(Abernathy, 2013 #287)  J Pain Symptom Manage | Cancer | Title Delivery strategies to optimize resource utilization and performance status for patients with advanced life-limiting illness: results from the "palliative care trial"   Location/Funding Australia/N.Z. Government, Non-govt/non-industry  Setting Clinic  Intervention Type Quality improvement  Unit of Analysis Patient  N Clusters 105 | Patient N enrolled: 461  Total Mean Age: 71 years  Sex % female: 50%  Race/Ethnicity: Aboriginal: <1%  Socioeconomic Status:  Level of Education:  Did not complete HS - 64%  Completed HS - 36% | Primary Outcomes:  PRO For Study 1 (case conferencing), area under the curve (AUC) for performance status using AKPS scale. For Study 2 and 3, usual pain intensity using Brief Pain Inventory. |
| Abramson, 2010(Abramson, 2010 #343)  Med J Aust | Chronic Lower Respiratory Diseases | Title Do spirometry and regular follow-up improve health outcomes in general practice patients with asthma or COPD? A cluster randomised controlled trial  Location/Funding Australia/N.Z. Government  Setting Clinic  Intervention Type Quality improvement  Unit of Analysis Patient  N Clusters 31 | Patient N enrolled: 305  Median Age: Arm 1: 54 years; Arm 2: 60 years; Arm3: 58 years  Sex % female: Arm 1: 71%; Arm 2: 62%; Arm 3: 66%  Race/Ethnicity: Unclear  Socioeconomic Status: Unclear | Primary Outcomes:  PRO Quality of life, Short Form (SF)-36 |
| Adams, 2015(Adams, 2015 #195)  Popul Health Manag | Cardiovascular Disease | Title Cluster-Randomized Trial of Clinical Pharmacist Tobacco Cessation Counseling Among Patients with Cardiovascular Disease  Location/Funding U.S. Unclear/NR  Setting Clinic  Intervention Type Behavioral  Unit of Analysis Clinical Pharmacist  N Clusters  NR | Patient N enrolled: 120  Total Mean Age: 65.7 years  Sex Total % female: 36.7%%  Race/Ethnicity: African-American: 4.2%; White: 80.8%  Socioeconomic Status:  Median family income=$61,613  Mean percent with at least some college education= 62.2% | Primary Outcomes:  Clinical The proportion of subjects who reported a tobacco cessation attempt |
| Al-sheyab, 2012(Al-sheyab, 2012 #1011)  Pediatrics | Chronic Lower Respiratory Diseases | Title Peer-led education for adolescents with asthma in Jordan: a cluster-randomized controlled trial  Location/Funding Middle East (includes Israel) Non-govt/non-industry  Setting School  Intervention Type The Adolescent Asthma Action (Triple A) program is a peer-led intervention to improve the health and well-being of students with asthma in a supportive school environment.  Unit of Analysis Patient  N Clusters 4 | Patient N enrolled: 261  Age: Unclear  Sex % female: Arm 1: 36.4%; Arm 2: 50.4%  Race/Ethnicity: Unclear  Socioeconomic Status: Unclear | Primary Outcomes:  PRO PAQLQ—Pediatric Asthma Quality of Life Questionnaire |
| Armour, 2013(Armour, 2013 #617)  J Asthma | Chronic Lower Respiratory Diseases | Title Feasibility and effectiveness of an evidence-based asthma service in Australian community pharmacies: a pragmatic cluster randomized trial  Location/Funding Australia/N.Z. Government  Setting Clinic  Intervention Type Quality improvement  Unit of Analysis Patient  N Clusters 106 | Patient N enrolled: 570  Age: Unclear  Sex % female: Unclear  Race/Ethnicity: Unclear  Socioeconomic Status: Unclear | Primary Outcomes:  PRO Asthma control at the completion of the 6-month service, measured by questionnaire and ACQ |
| Au, 2012(Au, 2012 #1176)  Chest | Chronic Lower Respiratory Diseases | Title A randomized trial to improve communication about end-of-life care among patients with COPD  Location/Funding U.S. Government  Setting Hospital  Intervention Type Quality improvement  Unit of Analysis Patient  N Clusters 92 | Patient N enrolled: 376  Mean Age: Arm 1: 69.4 years; Arm 2: 69.4 years  Sex % female: Arm 1: 2.1%; Arm 2: 3.8%  Race/Ethnicity: Arm 1 – White: 85.3%; Arm 2 – White: 87%  Socioeconomic Status:  Arm 1: More than high school education: 58.3%  Arm 2: More than high school education: 59.2% | Primary Outcomes:  PRO The previously validated quality of end-of-life communication score was our primary outcome measure (QOC) |
| Austin, 2010(Austin, 2010 #411)  BMJ | Chronic Lower Respiratory Diseases | Title Effect of high flow oxygen on mortality in chronic obstructive pulmonary disease patients in prehospital setting: randomised controlled trial  Location/Funding Australia/N.Z. Industry, Non-govt/non-industry  Setting EMS Setting  Intervention Type Quality improvement  Unit of Analysis Patient  N Clusters 62 | Patient N enrolled: 405  Mean Age: Arm 1: 69 years; Arm 2: 69 years  Sex % female: Arm 1: 50%; Arm 2: 54%  Race/Ethnicity: Unclear  Socioeconomic Status: Unclear | Primary Outcomes:  Mortality Pre-hospital and in-hospital mortality |
| Bell, 2010(Bell, 2010 #535)  Pediatrics | Chronic Lower Respiratory Diseases | Title Electronic health record-based decision support to improve asthma care: a cluster-randomized trial  Location/Funding U.S. Government  Setting Clinic  Intervention Type Quality improvement  Unit of Analysis Patient  N Clusters 12 | Patient N enrolled: 19,450  Age: Unclear  Sex % female: Unclear  Race/Ethnicity:  Arm 1: Hispanic - 5%; African-American - 5%; White - 80%; Other - 15%  Arm 2: Hispanic - 1%; African-American - 96%; White - 1%; Other - 3%  Arm 3: Hispanic - 3%; African-American - 50%; White - 40%; Other -10%  Arm 4: Hispanic - 3%; African-American - 80%; White - 30%; Other - 8%  Socioeconomic Status: Insurance, %  Arm 1: Commercial – 89%; Medicaid – 9%; Self-pay – 1%; Unknown – 1%  Arm 2: Commercial – 29%; Medicaid – 70%; Self-pay – 1%; Unknown – 1%  Arm 3: Commercial – 86%; Medicaid – 12%; Self-pay – 1%; Unknown – 1%  Arm 4: Commercial – 27%; Medicaid – 72%; Self-pay – 1%; Unknown – 1% | Primary Outcomes:  Clinical outcome 1. with persistent asthma with at least 1 prescription for a controller medication in each time period; 2. with persistent asthma with an uptodate ACP filed in the previous year; 3. aged 6 to 18 years with persistent asthma with documentation of spirometry performed |
| Benger, 2016(Benger, 2016 #1131)  Br J Anaesth | Cardiovascular Disease | Title Randomised comparison of the effectiveness of the laryngeal mask airway supreme, i-gel and current practice in the initial airway management of out of hospital cardiac arrest: a feasibility study  Location/Funding UK/Europe Unclear/NR  Setting Community  Intervention Type Devices  Unit of Analysis Patient  N Clusters 184 | Patient N enrolled: 615  Mean Age: Arm 1: 70 years; Arm 2: 71 years; Arm 3: 71 years  Sex Total % Male: Arm 1: 64.6%, Arm 2 61.2%; Arm 3 58.6%.  Race/Ethnicity: Unclear  Socioeconomic Status: Unclear | Primary Outcomes:  Process Study feasibility as measured by paramedic and patient recruitment, protocol adherence |
| Bergholdt, 2012(Bergholdt, 2012 #548)  BMJ Open | Cancer | Title Enhanced involvement of general practitioners in cancer rehabilitation: a randomised controlled trial  Location/Funding UK/Europe Government, Industry, Non-govt/non-industry  Setting Clinic, Hospital  Intervention Type Quality improvement  Unit of Analysis Patient  N Clusters 2181 | Patient N enrolled: 955  Mean Age: Arm 1: 63.6 years; Arm 2: 63.2 years  Sex % female: Arm 1: 71.4%; Arm 2: 72.6%  Race/Ethnicity: Unclear  Socioeconomic Status: Unclear | Primary Outcomes:  PRO Health-related quality of life measured 6 months after inclusion using the Global Health Status of the European Organization for Research and Treatment of Cancer Quality of Life Questionnaire Core 30 (EORTC QLQ-C30) |
| Berwanger, 2012(Berwanger, 2012 #385)  JAMA  BRIDGE-ACS | Cardiovascular Disease | Title Effect of a multifaceted intervention on use of evidence-based therapies in patients with acute coronary syndromes in Brazil: the BRIDGE-ACS randomized trial  Location/Funding Latin America Government, Non-govt/non-industry  Setting Hospital  Intervention Type Quality improvement  Unit of Analysis Patient  N Clusters 36 | Patient N enrolled: 618/555  Mean Age: Arm 1: 62 years; Arm 2: 62 years  Sex % female: Arm 1: 31.4%; Arm 2: 31.4%  Race/Ethnicity: Unclear  Socioeconomic Status: Unclear | Primary Outcomes:  Process Adherence to all eligible evidence-based therapies (aspirin; clopidogrel; anticoagulation with enoxaparin, unfractionated heparin, or fondaparinux; andstatins) during thefirst 24 hoursin patients without contraindications using the “all or none” approach. |
| Brotons, 2011(Brotons, 2011 #1149)  Rev Esp Cardiol  PREseAP | Cardiovascular Disease | Title Randomized clinical trial to assess the efficacy of a comprehensive programme of secondary prevention of cardiovascular disease in general practice: the PREseAP study  Location/Funding UK/Europe Government, Non-govt/non-industry  Setting Clinic, Health centers  Intervention Type Behavioral  Unit of Analysis Patient  N Clusters 42 | Patient N enrolled: 1224  Mean Age: Arm 1: 65.73 years; Arm 2: 67.19 years  Sex % female: Arm 1: 31.3%; Arm 2: 29%  Race/Ethnicity: Unclear  Socioeconomic Status: Level of Education, Employement Status  Occupational status: Arm 1: Employed: 16% Unemployed: 1.6% On sick leave or invalidity: 15.7%  Retired: 54.2% Other: 12.5%  Arm 2: Employed: 14.5% Unemployed: 2.5% On sick leave or invalidity: 11% Retired: 58.7% Other: 13.3%  Educational Status: Arm 1: Illiterate: 4.3%  No qualifications but able to read/write: 25.2%  Primary level: 46.7%  Secondary level: 12.4%  Higher educational qualification: 6.9% University diploma/degree: 4.5%   Arm 2: Illiterate: 4.3% No qualifications but able to read/ write: 34.7% Primary level: 39.8% Secondary level: 13.5% Higher educational qualification: 5.3% University diploma/degree: 2.3% | Primary Outcomes:  Clinical/Mortality The combination of total mortality and hospital cardiovascular readmissions during the study period (ischaemic cardiopathy, cardiac failure, stroke and peripheral vascular disease). |
| Casey, 2013(Casey, 2013 #453)  Thorax  PRINCE | Chronic Lower Respiratory Diseases | Title The effectiveness of a structured education pulmonary rehabilitation programme for improving the health status of people with moderate and severe chronic obstructive pulmonary disease in primary care: the PRINCE cluster randomised trial  Location/Funding UK/Europe Government, Industry  Setting Clinic  Intervention Type Behavioral, Quality improvement  Unit of Analysis Patient  N Clusters 32 | Patient N enrolled: 350  Mean Age: Arm 1: 68.8 yrs; Arm 2 68.4 yrs  Sex % female: Arm 1: 34.3%; Arm 2: 38.4%  Race/Ethnicity: Unclear  Socioeconomic Status:  Employment status  Paid work Employee: Arm 1: 9.6%; Arm 2: 7.0%  Paid work self-employed: Arm 1: 7.9%; Arm 2: 4.7%  Homemaker: Arm 1: 14.6%; Arm 2:  11.0%  Unemployed looking for work: Arm 1: 4.5%; Arm 2: 4.7%  Retired: Arm 1: 51.7%; Arm 2: 64.5%  Unable to work disability: Arm 1: 9.0%; Arm 2: 5.2%  Other: Arm 1: 2.8%; Arm 2: 2.9% | Primary Outcomes:  PRO The total score of the disease specific quality of life instrument, the Chronic Respiratory Questionnaire (CRQ) |
| Costantini, 2014(Costantini, 2014 #880)  Lancet | Cancer | Title Liverpool Care Pathway for patients with cancer in hospital: a cluster randomised trial  Location/Funding UK/Europe Government, Non-govt,non-industry  Setting Hospital  Intervention Type Quality improvement  Unit of Analysis Patient  N Clusters 16 | Patient N enrolled: 540  Mean Age: Arm 1: 75.6 years; Arm 2: 75.2 years  Sex % female: Arm 1: 39%; Arm 2: 38%  Race/Ethnicity: Unclear  Socioeconomic Status:  Level of Education  Arm 1:  <=5 yrs: 13%.  6-8: 30% >8: 57% Unk: N=28  Arm 2:  <=5 yrs: 10%.  6-8: 36% >8: 54% Unk: N=49 | Primary Outcomes:  PRO Mean score of the toolkit scale overall rating of patient-focused, family-centered care |
| Dear, 2012(Dear, 2012 #709)  Ann Oncol | Cancer | Title Impact of a cancer clinical trials web site on discussions about trial participation: a cluster randomized trial  Location/Funding Australia/N.Z. Government  Setting Clinic  Intervention Type Quality improvement  Unit of Analysis Patient  N Clusters 30 | Patient N enrolled: 494  Mean Age: Arm 1: 60 years; Arm 2: 59 years  Sex % female: Arm 1: 54%; Arm 2: 58%  Race/Ethnicity: Unclear  Socioeconomic Status: Unclear | Primary Outcomes:  Process The proportion of patients with whom participation in any clinical trial was discussed. |
| Du, 2014(Du, 2014 #689)  Circ Cardiovasc Qual Outcomes | Cardiovascular Disease | Title Hospital quality improvement initiative for patients with acute coronary syndromes in China: a cluster randomized, controlled trial  Location/Funding Asia Unclear/NR  Setting Hospital  Intervention Type Quality improvement  Unit of Analysis Patient  N Clusters 70 | Patient N enrolled: 15,141  Mean Age: Arm 1: 63.9 years; Arm 2: 64.4 years  Sex % female: Arm 1: 28.1%; Arm 2: 32.7%  Race/Ethnicity: Unclear  Socioeconomic Status:  Main Occupation  Manual 19.7%  Business 4.7%  Retired 43.5%  Annual Income  <10000 RMB (renminbi) 313 (22.9)  10-20000 RMB 604 (44.2)  21-30000 RMB 312 (22.8)  >30,000 RMB 137 (10.0)  Medical Insurance held 1370 (85.8)" "Main Occupation  Manual 404 (22.0)  Business 113 (6.2)  Retired 626 (34.1)  Annual Income  <10000 RMB (renminbi) 477 (27.4)  10-20000 RMB 639 (36.8)  21-30000 RMB 398 (22.9)  >30,000 RMB 224 (12.9)  Medical Insurance held 1472 (77.7) | Primary Outcomes:  Process 8 key performance indicators: Proportion of patients with final diagnosis c/w biomarker findings proportion of patients with STEMI receiving thrombosis or primary PCI among those arriving within 12 hours of symptoms onset door-to-needle time for patients with STEMI undergoing thrombolysis door-to-balloon time for patients with STEMI undergoing primary PCI proportion of high-risk patients undergoing coronary angiography proportion of low-risk patients (no ongoing symptoms, persistently normal ECG, and persistently normal biomarkers) undergoing functional testing proportion of patients discharged on combination medical therapy (including any anti platelet therapy, b-blocker, ACE-I or ARB, and statin. hospital length of stay |
| Fihn, 2011(Fihn, 2011 #207)  Arch Intern Med | Cardiovascular Disease | Title Collaborative care intervention for stable ischemic heart disease  Location/Funding U.S. Government  Setting Clinic  Intervention Type Quality improvement  Unit of Analysis Patient  N Clusters 283 | Patient N enrolled: 703  Mean Age: Arm 1: 68.3 years; Arm 2: 67.2 years  Sex % female: Arm 1: 1.2%; Arm 2: 3.3%  Race/Ethnicity: White Arm 1 – 79.4%; White Arm 2 – 80.8%  Socioeconomic Status:  Education:  Arm 1:  > High School education: 72.1%  Arm 2:  >High School education: 78.3% | Primary Outcomes:  PRO Change in the SAQ anginal frequency score |
| Flather, 2011(Flather, 2011 #199)  Am Heart J  EQUIP-ACS | Cardiovascular Disease | Title Cluster-randomized trial to evaluate the effects of a quality improvement program on management of non-ST-elevation acute coronary syndromes: The European Quality Improvement Programme for Acute Coronary Syndromes (EQUIP-ACS)  Location/Funding UK/Europe Unclear/NR  Setting Hospital  Intervention Type Quality improvement  Unit of Analysis Patient  N Clusters 38 | Patient N enrolled: 2604  Age: Arm 1: 65.8 years; Arm 2: 66.1 years; Arm 3: 65.1 years; Arm 4: 65.6 years  Sex % female: Arm 1: 27.6%; Arm 2: 27.2%; Arm 3: 31.9%; Arm 4: 32.8%  Race/Ethnicity: Unclear  Socioeconomic Status: Unclear | Primary Outcomes:  Process  Composite of 8 strategies: (1) risk stratification according to a recognized algorithm; (2) coronary angiography 72 hours in medium- to high-risk patients; (3) in-hospital anticoagulation; (4) β-blockers in patients with heart failure; (5) statins; (6) angiotensin-converting enzyme (ACE) inhibitors in patients with heart failure, hypertension, diabetes, or renal impairment; (7) clopidogrel loading dose; and (8) clopidogrel at discharge. |
| Foster, 2014(Foster, 2014 #821)  J Allergy Clin Immunol | Chronic Lower Respiratory Diseases | Title Inhaler reminders improve adherence with controller treatment in primary care patients with asthma  Location/Funding Australia/N.Z. Government  Setting Clinic  Intervention Type Behavioral  Unit of Analysis Patient  N Clusters 43 | Patient N enrolled: 143  Age: 40.3 years  Sex % female: 62%  Race/Ethnicity: Unclear  Socioeconomic Status:  Highest Level of Education: high school or lower  34% | Primary Outcomes:  PRO The Asthma Control Test (ACT) score (assessed at the patient level). |
| Garbutt, 2015(Garbutt, 2015 #198)  J Allergy Clin Immunol | Chronic Lower Respiratory Diseases | Title A cluster-randomized trial shows telephone peer coaching for parents reduces children's asthma morbidity  Location/Funding U.S. Government  Setting Clinic  Intervention Type Behavioral  Unit of Analysis Patient, Other  N Clusters 22 | Family N enrolled: 948  Mother Age: Unclear/Not Reported  Mother Race/Ethnicity:  Arm 1: White 53%; African-American 42.9%; Other 4.1%, Hispanic 0.4%  Arm 1: White 78.2%; African-American 18.9%%; Other 2.9%, Hispanic 3.3%  Mother Socioeconomic Status:  Income  <$10,000 Arm 1 10.6%; Arm 2 6.5%  10,000-24,999 Arm 1 10.9%; Arm 2 7.6%  25,000-49,999 Arm 1 20.1%; Arm 2 16.4%  50,000-74,999 Arm 1 14.9%; Arm 2 18.5%  75,000-99,999 Arm 1 13.6%; Arm 2 14.5%  >100,000 Arm 1 29.9%; Arm 2 36.2%  Mother College Graduation: Arm 1 55.8%; Arm 2 78.2%  Child Mean Age: Arm 1 6.6 years; Arm 2 7.1 years  Child Male Sex: Arm 1 63.9%; Arm 2 61.5%  Child Race/Ethnicity: Not Reported  Child Insurance:  Medicare: Arm 1 26.8%; Arm 2 20.5% | Primary Outcomes:  Clinical Symptom-free days for the child |
| Garcia-Cardenas, 2013(Garcia-Cardenas, 2013 #388)  Respir Med | Chronic Lower Respiratory Diseases | Title Effect of a pharmacist intervention on asthma control. A cluster randomised trial  Location/Funding UK/Europe Industry  Setting Pharmacies  Intervention Type Behavioral  Unit of Analysis Patient  N Clusters 51 | Patient N enrolled: 373  Total Mean Age: 55.8 years  Sex % female: 53.9%  Race/Ethnicity: Unclear  Socioeconomic Status:  No education: 14.9%  Primary: 36.7%  Secondary/Vocational education: 25.7%  University: 22.7%  Employment :  Unpaid worker: 30.7%  Paid worker: 33.3%  Unemployed or retired person: 36% | Primary Outcomes:  PRO Asthma control, assessed by Asthma Control Questionnaire (ACQ) |
| Guldbrandt, 2015(Guldbrandt, 2015 #402)  BMC Cancer | Cancer | Title The effect of direct access to CT scan in early lung cancer detection: an unblinded, cluster-randomised trial  Location/Funding UK/Europe Government, Industry, Non-govt/non-industry  Setting Clinic  Intervention Type Quality improvement  Unit of Analysis Patient  N Clusters 119 | Patient N enrolled: 331  Total Mean Age: 69.4 years  Sex Total % female: 52.6%  Race/Ethnicity: Unclear  Socioeconomic Status:  Education  <10 yrs – 41.1%  10–15 yrs - 44.1%  >15 yrs - 12.1 yrs  Unknown - 2.7 yrs | Primary Outcomes:  Process The primary care interval (time from patient's first presentation in primary care until referral to secondary care) |
| Hilberink, 2011(Hilberink, 2011 #650)  Patient Educ Couns | Chronic Lower Respiratory Diseases | Title General practice counseling for patients with chronic obstructive pulmonary disease to quit smoking: impact after 1 year of two complex interventions  Location/Funding UK/Europe Unclear/NR  Setting Clinic  Intervention Type Behavioral  Unit of Analysis Patient  N Clusters 68 | Patient N enrolled: 667  Mean Age: Arm 1: 60.1 years; Arm 2: 58 years; Arm 3: 60.7 years  Sex Total % female: Arm 1: 44.6%; Arm 2: 53.5%; Arm 3: 52.2%  Race/Ethnicity: Unclear  Socioeconomic Status:  Education  Arm 1:  Primary: 48%  Secondary: 38.5%  Advanced: 7.4%  Arm 2:  Primary: 47.7%  Secondary: 41.6%  Advanced: 7.4%  Arm 3:  Primary: 47.1%  Secondary: 41.7%  Advanced: 6.2% | Primary Outcomes:  Clinical Biochemically verified point prevelance |
| Holton, 2011(Holton, 2011 #357)  Int J Qual Health Care | Chronic Lower Respiratory Diseases | Title Does spirometry training in general practice improve quality and outcomes of asthma care?  Location/Funding Australia/N.Z. Government  Setting Clinic  Intervention Type Quality improvement  Unit of Analysis Patient  N Clusters 40 | Patient N enrolled: 397  Total Mean Age: 56.7 years  Sex Total % female: 67.5%  Race/Ethnicity: Unclear  Socioeconomic Status:  Education  Bachelor degree or higher 14.6%  Post-school qualifications 36%  Primary or secondary schooling 49.4%  Socio-economic index  Low or low/medium 49.4%  Medium/high or high 50.6% | Primary Outcomes:  PRO Health-related quality of life measured by Juniper's Mini Asthma Quality of Life Questionnaire |
| Holton, 2010(Holton, 2010 #1326)  Med J Aust | Chronic Lower Respiratory Diseases | Title Systematic care for asthma in Australian general practice: a randomised controlled trial  Location/Funding Australia/N.Z. Government  Setting Clinic  Intervention Type Quality improvement  Unit of Analysis Patient  N Clusters 40 | Patient N enrolled: 565  Total Mean Age: 58 years  Sex Total % female: 63.5%  Race/Ethnicity: Unclear  Socioeconomic Status:  Socioeconomic index, based on postcode Low or low–medium: 346 (61.2%)  Medium–high or high: 218 (38.6%)  Missing data: 16 (0.3%)  Educational qualifications High school only: 303 (53.6%)  Trade, or postsecondary certificate: 110 (19.5%) Diploma, degree or higher: Total: 152 (26.9%)  Employment status Employed: Control: 205 (36.2%)  Retired: 250 (44.3%) Home duties or other: 110 (19.5%) | Primary Outcomes:  Primary outcome not clearly stated. |
| Honkoop, 2015(Honkoop, 2015 #1322)  J Allergy Clin Immunol | Chronic Lower Respiratory Diseases | Title Symptom- and fraction of exhaled nitric oxide-driven strategies for asthma control: A cluster-randomized trial in primary care  Location/Funding UK/Europe Non-govt/non-industry  Setting Clinic  Intervention Type Asthma control targets  Unit of Analysis Patient  N Clusters 131 | Patient N enrolled: 647  Mean Age: Arm 1: 38.9 years; Arm 2: 39.9 years; Arm 3: 39.5  Sex % female: Arm 1: 68.4%; Arm 2: 65.8%; Arm 3: 72.3%  Race/Ethnicity: Unclear  Socioeconomic Status: Unclear | Primary Outcomes:  Health economics societal costs per quality-adjusted life year (QALY) gained |
| Hopkinson, 2010(Hopkinson, 2010 #285)  J Pain Symptom Manage | Cancer | Title The deliverability, acceptability, and perceived effect of the Macmillan approach to weight loss and eating difficulties: a phase II, cluster-randomized, exploratory trial of a psychosocial intervention for weight- and eating-related distress in people with advanced cancer  Location/Funding UK/Europe Non-govt/non-industry  Setting Clinic  Intervention Type Behavioral  Unit of Analysis Patient  N Clusters 2 | Patient N enrolled: 65  Total Mean Age: 69 years  Sex Total % female: 76%  Race/Ethnicity: Unclear  Socioeconomic Status: Unclear | Primary Outcomes:  PRO 1) Self-reported eating related distress (ERD), assessed by a visual analog scale 2) self-report weight related distress (WRD), assessed by VAS |
| Hostler, 2011(Hostler, 2011 #425)  BMJ  PARAMEDIC | Cardiovascular Disease | Title Effect of real-time feedback during cardiopulmonary resuscitation outside hospital: prospective, cluster-randomised trial  Location/Funding U.S., Canada Government, Non-govt/non-industry  Setting EMS Setting  Intervention Type Quality improvement  Unit of Analysis Cluster- EMS agency or vehicle  N Clusters 39 | Patient N enrolled: 1,586  Mean Age: Arm 1: 66 years; Arm 2: 65 years  Sex % female: Arm 1: 38%; Arm 2: 36%  Race/Ethnicity: Unclear  Socioeconomic Status: Unclear | Primary Outcomes:  Survival Pre-hospital return of circulation before hospital admission |
| Jahn, 2014(Jahn, 2014 #767)  Pain | Cancer | Title Improvement of pain-related self-management for cancer patients through a modular transitional nursing intervention: a cluster-randomized multicenter trial  Location/Funding UK/Europe Government  Setting Hospital  Intervention Type Behavioral, Quality improvement  Unit of Analysis Patient  N Clusters 18 | Patient N enrolled: 263  Mean Age: Arm 1: 55.9 years; Arm 2: 57.75 years  Sex % female: Arm 1: 42.9%; Arm 2: 42.2%  Race/Ethnicity: Unclear  Socioeconomic Status: Unclear | Primary Outcomes:  PRO 1) Barriers Questionnaire – BQ II) 2) The effectiveness of the SCION-PAIN program was primarily assessed by the difference in patient-related barriers to management of cancer pain between the control and intervention groups using the Barriers Questionnaire II (BQ II) |
| Janson, 2010(Janson, 2010 #970)  Chest | Chronic Lower Respiratory Diseases | Title Objective airway monitoring improves asthma control in the cold and flu season: a cluster randomized trial  Location/Funding U.S. Government  Setting Clinical laboratory  Intervention Type Objective airway monitoring by providing peak flow graphs  Unit of Analysis Provider  N Clusters 43 | Patient N enrolled: 139  Mean Age: Arm 1: 49.7 years; Arm 2: 50.3 years  Sex % female: Arm 1: 74%; Arm 2: 63%  Race/Ethnicity:  Arm 1: African-American - 21%; Hispanic - 6%; Asian - 12%; Hawaiian - 0%; White - 60%; Multiracial - 7%  Arm 2: African-American - 16%; Hispanic - 6%; Asian - 18%; Hawaiian - 1%; White - 59%; Multiracial - 6%  Socioeconomic Status:  Insurance Status  Arm 1: Insured – 98%  Arm 2: Insured – 98% | Primary Outcomes:  Clinical Inhaled corticosteroid adherence |
| Jiang, 2015(Jiang, 2015 #720)  Genet Mol Res | Chronic Lower Respiratory Diseases | Title Impact of adherence to GOLD guidelines on 6-minute walk distance, MRC dyspnea scale score, lung function decline, quality of life, and quality-adjusted life years in a Shanghai suburb  Location/Funding Asia Unclear/NR  Setting Clinic  Intervention Type Quality improvement  Unit of Analysis Patient  N Clusters NR | Patient N enrolled: 132  Mean Age: Arm 1: 69 years; Arm 2: 71 years  Sex % female: Arm 1: 50.8%; Arm 2: 37.6%  Race/Ethnicity: Unclear  Socioeconomic Status: Unclear | Primary Outcomes:  Primary outcome not clearly stated. |
| Kinsman, 2012(Kinsman, 2012 #337)  Aust J Rural Health | Cardiovascular Disease | Title Do clinical pathways enhance access to evidence-based acute myocardial infarction treatment in rural emergency departments?  Location/Funding Australia/N.Z. Non-govt/non-industry  Setting Hospital  Intervention Type Quality improvement  Unit of Analysis Patient  N Clusters 6 | Patient N enrolled: 108  Mean Age: Arm 1: 63.5 years; Arm 2: 64.7 years; Arm 3: 64.2 years; Arm 4: 57.6 years  Sex % female: Arm 1: 20%; Arm 2: 28%; Arm 3: 19%; Arm 4: 8%  Race/Ethnicity: Unclear  Socioeconomic Status: Unclear | Primary Outcomes:  Process  The proportion of eligible AMI patients receiving a thrombolytic drug |
| Kruis, 2014(Kruis, 2014 #466)  BMJ  RECODE | Chronic Lower Respiratory Diseases | Title Effectiveness of integrated disease management for primary care chronic obstructive pulmonary disease patients: results of cluster randomised trial  Location/Funding UK/Europe Government, Industry  Setting Clinic  Intervention Type Quality improvement  Unit of Analysis Patient  N Clusters 40 | Patient N enrolled: 1,086  Mean Age: Arm 1: 68.2 years; Arm 2: 68.4 years  Sex % female: Arm 1: 49.4%; Arm 2: 42.4%  Race/Ethnicity: Unclear  Socioeconomic Status:  Insurance Status  Arm 1:  Low education – 39.2%  Employment – 27.7%  Arm 2:  Low education – 41.5%  Employment – 28.8% | Primary Outcomes:  PRO Change in health related quality of life on the Clinical COPD Questionnaire (CCQ) |
| Krum, 2013(Krum, 2013 #1338)  Cardiovasc Ther | Cardiovascular Disease | Title Telephone support to rural and remote patients with heart failure: the Chronic Heart Failure Assessment by Telephone (CHAT) study  Location/Funding Australia/N.Z. Government, Non-govt/non-industry  Setting Clinic  Intervention Type Telephone support  Unit of Analysis Patient  N Clusters 127 | Patient N enrolled: 405  Mean Age: Arm 1: 73 years; Arm 2: 73 years  Sex % female: Arm 1: 36%; Arm 2: 38%  Race/Ethnicity: Unclear  Socioeconomic Status: Unclear | Primary Outcomes:  Clinical/Mortality/PRO The primary endpoint of the study was the Packer clinical composite score at 12 months comprising the following elements: 1 Death. 2 Hospital admission for heart failure. 3 Withdrawal from study due to worsening heart failure. 4 Seven-point global health assessment questionnaire with regard to overall well-being in comparison with baseline. |
| Levine, 2011(Levine, 2011 #779)  Arch Intern Med | Cardiovascular Disease | Title Improving care after myocardial infarction using a 2-year internet-delivered intervention: the Department of Veterans Affairs myocardial infarction-plus cluster-randomized trial  Location/Funding U.S. Government  Setting Clinic  Intervention Type Quality improvement  Unit of Analysis Patient  N Clusters 168 | Patient N enrolled: 15,847  Percent in Each Age Category:  <55 yrs: Arm 1: 16.9%; Arm 2: 15.0%; Arm 3: 10.6%; Arm 4: 9.0%  55-64: Arm 1: 33.7%; Arm 2: 35.7%; Arm 3: 37.9%; Arm 4: 37.9%  65-74: Arm 1: 25.4%; Arm 2: 26.1%; Arm 3: 25.1%; Arm 4: 26.7%  >75 yrs: Arm 1: 24.0%; Arm 2: 23.2%; Arm 3: 26.5%; Arm 4: 26.4%  Sex % Male: Arm 1: 98.6%; Arm 2: 98.3%; Arm 3: 98.8%; Arm 4: 98.7%  Race/Ethnicity:  Arm 1: White 68.2%; Black 9.7%; Other 1.7%; Unknown 20.3%  Arm 2: White 69.4%; Black 8.1%; Other 2.3%; Unknown 20.3%  Arm 3: White 66.0%; Black 8.7%; Other 1.5%; Unknown 23.8%  Arm 4: White 70.6%; Black 8.0%; Other 1.6%; Unknown 19.9%  Socioeconomic Status:  Unclear/Not Reported | Primary Outcomes:  Process Percentage of patients who achieved each of the 7 clinical indicators:  Subgroups:  NR |
| Livingston, 2015(Livingston, 2015 #484)  Cancer  ENGAGE | Cancer | Title Effects of a clinician referral and exercise program for men who have completed active treatment for prostate cancer: A multicenter cluster randomized controlled trial (ENGAGE)  Location/Funding Australia/N.Z. Unclear/NR  Setting Clinic  Intervention Type Behavioral  Unit of Analysis Patient  N Clusters 13 | Patient N enrolled: 147  Total Mean Age: 64.7 years  Sex Total % female: 0  Race/Ethnicity: Unclear  Socioeconomic Status:  Arm 1:  Education:  Primary: 6.7%  Secondary: 34.4%  Certificate/diploma: 33.3%  University: 26.9%  Private Health Service: 25 (26.9)  Arm 2:  Education status, 5.7%  Secondary 28.3%  Certificate/diploma 35.9%  University 30.1%  Private health service 14 (25.9) | Primary Outcomes:  PRO Self-reported number of moderate-vigorous exercise activity |
| Lowrie, 2012(Lowrie, 2012 #1022)  Eur Heart J  HOOPS | Cardiovascular Disease | Title Pharmacist intervention in primary care to improve outcomes in patients with left ventricular systolic dysfunction  Location/Funding UK/Europe Government  Setting Clinic  Intervention Type Pharmacist intervention  Unit of Analysis Patient  N Clusters 174 | Patient N enrolled: 2,164  Mean Age: Arm 1: 70.6 years; Arm 2: 70.6 years  Sex % female: Arm 1: 29%; Arm 2: 31%  Race/Ethnicity: Unclear  Socioeconomic Status: Unclear | Primary Outcomes:  Mortality Composite of death from any cause or hospital admission for worsening heart failure, analysed as time to first event. |
| McCorkie, 2015(McCorkie, 2015 #25)  J Palliat Med | Cancer | Title An Advanced Practice Nurse Coordinated Multidisciplinary Intervention for Patients with Late-Stage Cancer: A Cluster Randomized Trial  Location/Funding U.S. Unclear/NR  Setting Clinic  Intervention Type Quality improvement  Unit of Analysis Patient  N Clusters 4 | Patient N enrolled: 146  Total Age:  < 65 years: 91 (62.3%)  Age 65 yrs and older: 55 (37.7%)"  Sex Total % female: 56.2%  Race/Ethnicity: Total White – 84.9%; Total Other: 15.1%  Socioeconomic Status:  Education  High school or less: 28.8%  College or more: 71.2% | Primary Outcomes:  PRO 5 primary outcomes: Symptoms (symptom distress scale) Health Distress (health distress scale) Depression (PHQ-9) Functional status (enforced social dependency scale) Self-reported health (part of the SF-12) |
| Meng, 2016(Meng, 2016 #718)  Patient Educ Couns | Cardiovascular Disease | Title The impact of a self-management patient education program for patients with chronic heart failure undergoing inpatient cardiac rehabilitation  Location/Funding UK/Europe Unclear/NR  Setting Rehabilitation clinic  Intervention Type Behavioral  Unit of Analysis Patient  N Clusters 98 | Patient N enrolled: 475  Mean Age: Arm 1: 61.9 years; Arm 2: 61.2 years  Sex % female: Arm 1: 19.8%; Arm 2: 25%  Race/Ethnicity: Unclear  Socioeconomic Status:  Education,  Less than Junior (<10 y; basic secondary school): Arm 1: 54.7%; Arm 2: 47.9%  Junior (10 y: middle-level secondary school): Arm 1: 23.1%; Arm 2: 25.4%  Senior (high school graduate): Arm 1: 20%; Arm 2: 24.2%  Other: Arm 1: 2.2%; Arm 2: 2.5%  Employment Status:  Employed: Arm 1: 40.4%; Arm 2: 39.3%  Retired: Arm 1: 43.6%; Arm 2: 45.7%  Unemployed: Arm 1: 7.6%; Arm 2: 8.5%  Other: Arm 1: 8.4%; Arm 2: 6.5% | Primary Outcomes:  PRO Self-management competence (self-monitoring and insight, skill and technique acquisition, self-efficacy) |
| Nichol, 2015(Nichol, 2015 #1370)  N Engl J Med | Cardiovascular Disease | Title Trial of Continuous or Interrupted Chest Compressions during CPR  Location/Funding U.S., Canada Government  Setting Out-of-hospital EMS  Intervention Type Continuous vs. interrupted chest compressions  Unit of Analysis Patient  N Clusters 47 | Patient N enrolled: 26,148  Mean Age: Arm 1: 66.4 years; Arm 2: 66.2 years  Sex % female: Arm 1: 36.5%; Arm 2: 35.6%  Race/Ethnicity: Unclear  Socioeconomic Status: Unclear | Primary Outcomes:  Survival Rate of survival to hospital discharge |
| Nicolaije, 2015(Nicolaije, 2015 #721)  J Clin Oncol | Cancer | Title Impact of an Automatically Generated Cancer Survivorship Care Plan on Patient-Reported Outcomes in Routine Clinical Practice: Longitudinal Outcomes of a Pragmatic, Cluster Randomized Trial  Location/Funding UK/Europe Unclear/NR  Setting Hospital  Intervention Type Quality improvement  Unit of Analysis Patient  N Clusters 12 | Patient N enrolled: 221  Total Mean Age: 67.4 years  Sex % female: 100%  Race/Ethnicity: Unclear  Socioeconomic Status:  Arm 1:  High 14% Intermediate 60% Low 25%  Arm 2:  High 7% Intermediate 74% Low 19% | Primary Outcomes:  PRO Satisfaction with information as measured by Cancer QOL scale. |
| Nokela, 2010(Nokela, 2010 #815)  Respiration | Chronic Lower Respiratory Diseases | Title The influence of structured information and monitoring on the outcome of asthma treatment in primary care: a cluster randomized study  Location/Funding UK/Europe Government, Non-govt/non-industry  Setting Clinic  Intervention Type Quality improvement  Unit of Analysis Patient  N Clusters 19 | Patient N enrolled: 141  Mean Age: Arm 1: 48 years; Arm 2: 52.2 years  Sex % female: Arm 1: 75%; Arm 2: 64.9%  Race/Ethnicity: Unclear  Socioeconomic Status: Unclear | Primary Outcomes:  PRO The change in the score of the asthma control questionnaire (ACQ) between the 2 visits in the study. |
| Ono, 2015(Ono, 2015 #1259)  Am J Emerg Med | Chronic Lower Respiratory Diseases | Title Should laryngeal tubes or masks be used for out-of-hospital cardiac arrest patients?  Location/Funding Asia Unclear/NR  Setting EMS Setting  Intervention Type Devices  Unit of Analysis Patient  N Clusters 14 | Patient N enrolled: 313  Mean Age: Arm 1: 72.41 years; Arm 2: 75.84 years  Sex % female: Arm 1: 39.2%; Arm 2: 38.8%  Race/Ethnicity: Unclear  Socioeconomic Status: Unclear | Primary Outcomes:  Process The time from CPR initiation to device insertion |
| Overbeek, 2010(Overbeek, 2010 #541)  Virchows Arch | Cancer | Title Electronic reminders for pathologists promote recognition of patients at risk for Lynch syndrome: cluster-randomised controlled trial  Location/Funding UK/Europe Government  Setting Pathology Laboratories  Intervention Type Quality improvement  Unit of Analysis Patient  N Clusters 12 | Patient N enrolled: 256  Age at Diagnosis: Arm 1: 48.2 years, Arm 2: 46.2 years  Sex % female: Unclear  Race/Ethnicity: Unclear  Socioeconomic Status: Unclear | Primary Outcomes:  Clinical The percentage of patients recognized to be at risk for Lynch syndrome. |
| Perkins, 2015(Perkins, 2015 #902)  Lancet  PARAMEDIC | Cardiovascular Disease | Title Mechanical versus manual chest compression for out-of-hospital cardiac arrest (PARAMEDIC): a pragmatic, cluster randomised controlled trial  Location/Funding UK/Europe Government  Setting Out of Hospital EMS  Intervention Type Devices  Unit of Analysis Patient  N Clusters 418 | Patient N enrolled: 4,471  Mean Age: Arm 1: 71 years; Arm 2: 71.6 years  Sex % female: Arm 1: 37%; Arm 2: 37%  Race/Ethnicity: Unclear  Socioeconomic Status: Unclear | Primary Outcomes:  Survival Survival to 30 days after the cardiac arrest event |
| Rixon, 2015(Rixon, 2015 #1192)  Clin Respir J | Chronic Lower Respiratory Diseases | Title A RCT of telehealth for COPD patient's quality of life: the whole system demonstrator evaluation  Location/Funding UK/Europe Unclear/NR  Setting Clinic  Intervention Type Telehealth  Unit of Analysis Patient  N Clusters 121 | Patient N enrolled: 578  Total Mean Age: 71.79 years  Sex Total % female: 43.5%  Race/Ethnicity: Unclear  Socioeconomic Status:  Level of education score:  0=no formal education, 1=GCSE/O’levels, 2=A’levels/HNC, 3=University level and 4=Graduate/Professional.  Mean 0.70; SE 0.046 | Primary Outcomes:  Primary outcome not clearly stated. |
| Schwalm, 2015(Schwalm, 2015 #162)  Am Heart J  DERLA-STEMI | Cardiovascular Disease | Title Cluster randomized controlled trial of Delayed Educational Reminders for Long-term Medication Adherence in ST-Elevation Myocardial Infarction (DERLA-STEMI)  Location/Funding Canada Non-govt/non-industry  Setting Hospital  Intervention Type Behavioral  Unit of Analysis Patient  N Clusters 582 | Patient N enrolled: 888  Mean Age: Arm 1: 63.3 years; Arm 2: 62.4 years  Sex % female: Arm 1: 31.4%; Arm 2: 26.9%  Race/Ethnicity: Unclear  Socioeconomic Status: Unclear | Primary Outcomes:  Clinical Proportion of participants who describe taking a statin, BB, angiotensin blocker (ACEI or ARD), and acetyalsalicylic acid (ASA). (4 of 4 medication classes) |
| Simunovic, 2010(Simunovic, 2010 #191)  CMAJ | Cancer | Title The cluster-randomized Quality Initiative in Rectal Cancer trial: evaluating a quality-improvement strategy in surgery  Location/Funding Canada Unclear/NR  Setting Hospital  Intervention Type Quality improvement  Unit of Analysis Patient  N Clusters 16 | Patient N enrolled: 1,015  Median Age: Arm 1: 69 years; Arm 2: 68 years  Sex % female: Arm 1: 32.2%; Arm 2: 35.9%  Race/Ethnicity: Unclear  Socioeconomic Status: Unclear | Primary Outcomes:  Clinical Hospital rates of permanent colostomy |
| Smidth, 2013(Smidth, 2013 #394)  BMC Health Serv Res | Chronic Lower Respiratory Diseases | Title The effect of an active implementation of a disease management programme for chronic obstructive pulmonary disease on healthcare utilization--a cluster-randomised controlled trial  Location/Funding UK/Europe Government, Non-govt/non-industry  Setting Clinic  Intervention Type Behavioral  Unit of Analysis Patient  N Clusters 9/7 | Patient N enrolled: 1372  Total Mean Age: 66.9 years  Sex % female: 51.5%.  Race/Ethnicity: Unclear  Socioeconomic Status: Unclear | Primary Outcomes:  Process Adherence to the disease management programme in the GP practices measured by specific services of planned and additional preventive consultations, and the number of spirometries |
| Smidth, 2013(Smidth, 2013 #1003)  BMC Fam Pract | Chronic Lower Respiratory Diseases | Title Patient-experienced effect of an active implementation of a disease management programme for COPD - a randomised trial  Location/Funding UK/Europe Government, Non-govt/non-industry  Setting Clinic  Intervention Type Disease Management Program  Unit of Analysis Patient  N Clusters 38 | Patient N enrolled: 443  Total Mean Age: Arm 1: 68.3 years; Arm 2: 66.5 years  Sex % Total female: Arm 1: 51.7%; Arm 2: 51.9%  Race/Ethnicity: Unclear  Socioeconomic Status: Unclear | Primary Outcomes:  PRO PACIC score |
| Smith, 2012(Smith, 2012 #58)  Thorax  ARRISA | Chronic Lower Respiratory Diseases | Title The at-risk registers in severe asthma (ARRISA) study: a cluster-randomised controlled trial examining effectiveness and costs in primary care  Location/Funding UK/Europe Unclear/NR  Setting Clinic  Intervention Type Quality improvement  Unit of Analysis Patient  N Clusters 30 | Patient N enrolled: 969  Total Mean Age: 45.5 years  Sex % Total female: 61.3%  Race/Ethnicity: Unclear  Socioeconomic Status: Unclear | Primary Outcomes:  Clinical Number of patients experiencing a moderate-severe exacerbation |
| Steill, 2011(Steill, 2011 #370)  N Engl J Med | Cardiovascular Disease | Title Early versus later rhythm analysis in patients with out-of-hospital cardiac arrest  Location/Funding U.S., Canada Government, Non-govt/non-industry  Setting EMS Setting  Intervention Type Quality improvement  Unit of Analysis Patient  N Clusters 20 | Patient N enrolled: 10,365  Mean Age: Arm 1: 66.7 yrs; Arm 2: 66.7 yrs  Sex % female: Arm 1: 35.6%; Arm 2: 36.1%  Race/Ethnicity: Unclear  Socioeconomic Status: Unclear | Primary Outcomes:  Mortality Survival to hospital discharge with satisfactory functional status, defined as a score of 3 or less on the modified Rankin scale |
| Stout, 2012(Stout, 2012 #873)  Acad Pediatr | Chronic Lower Respiratory Diseases | Title Learning from a distance: effectiveness of online spirometry training in improving asthma care  Location/Funding U.S. Government  Setting Clinic  Intervention Type Quality improvement  Unit of Analysis Clinic  N Clusters 16 | Patient N enrolled: Unclear  Age: Unclear  Sex % female: Unclear  Race/Ethnicity: Unclear  Socioeconomic Status: Unclear | Primary Outcomes:  Clinical Spirometry test frequency and the percentage with acceptable quality |
| Strasser, 2016(Strasser, 2016 #424)  Ann Oncol  Other : SAKK 95/06 | Cancer | Title The effect of real-time electronic monitoring of patient-reported symptoms and clinical syndromes in outpatient workflow of medical oncologists: E-MOSAIC, a multicenter cluster-randomized phase III study (SAKK 95/06)  Location/Funding UK/Europe Government, Industry, Non-govt/non-industry  Setting Clinic  Intervention Type Quality improvement  Unit of Analysis Patient  N Clusters 82 | Patient N enrolled: 264  Median Age: Arm 1: 67.3 years; Arm 2: 65.1 years  Sex % female: Arm 1: 39%; Arm 2: 35%  Race/Ethnicity: Unclear  Socioeconomic Status:  Education  Arm 1:  Basic education - 79%  Additional education - 20%  Missing - 1%  Arm 2:  Basic education - 72%  Additional education - 28%  Missing - 1% | Primary Outcomes:  PRO Change in Global Quality of Life (G-QoL), |
| Sulaiman, 2010(Sulaiman, 2010 #342)  Fam Pract | Chronic Lower Respiratory Diseases | Title Do small group workshops and locally adapted guidelines improve asthma patients' health outcomes? A cluster randomized controlled trial  Location/Funding Australia/N.Z. Government, Industry  Setting Clinic  Intervention Type Behavioral, Quality improvement  Unit of Analysis Patient  N Clusters 29 | Patient N enrolled: 411  Age:  Arm 1: 2–6 years - 42.3%; 7–10 years - 29.5%; 11–14 years - 28.2%  Arm 2: 2–6 years - 37.3%; 7-10 years - 38.1%; 11–14 years 24.6%  Arm 3: 2–6 years - 44.6%; 7–10 years - 34.7%; 11–14 years - 20.7%  Sex % female: Arm 1: 35.7%; Arm 2: 36.2%; Arm 3: 40.8%  Race/Ethnicity: Unclear  Socioeconomic Status:  Total household income before tax per year  Arm 1:  <A$30 000 - 33.3%  >A$300 000 - 66.7%  Arm 2:  <A$30 000 – 25.8%  >A$300 000 – 74.2%  Arm 3:  <A$30 000 – 35.1%  >A$300 000 – 64.9%  Employed in the past 12 months  Arm 1:  Yes - 64.3%  No - 35.7%  Arm 2:  Yes – 63.8%  No – 36.2%  Arm 3:  Yes – 87.8%  No – 12.2% | Primary Outcomes:  PRO Change in ownership of a written asthma action plan (WAAP) |
| Tamblyn, 2015(Tamblyn, 2015 #568)  J Am Med Inform Assoc | Chronic Lower Respiratory Diseases | Title Evaluating the impact of an integrated computer-based decision support with person-centered analytics for the management of asthma in primary care: a randomized controlled trial  Location/Funding Canada Government, Non-govt/non-industry  Setting Clinic  Intervention Type Quality improvement  Unit of Analysis Patient  N Clusters 81 | Patient N enrolled: 4,447  Mean Age:  Arm 1:  Age at entry (Years)  5–18: 2.7%  19–45: 27.3%  46–65: 36.1%  >65: 33.9%  Arm 2:  Age at entry (Years)  5–18: 5.5%  19–45: 27.9%  46–65: 35.3%  >65: 31.3%  Sex % female: Arm 1: 67% Arm 2: 67.8%  Race/Ethnicity: Unclear  Socioeconomic Status:  Income Level  Arm 1: Income – Mean $45,103  Arm 2: Income – Mean $45,807 | Primary Outcomes:  Clinical Rate of out of control asthma episodes defined as excessive use of inhalers, ER visit, or hospitalization for asthma |
| van Bragt, 2015(van Bragt, 2015 #1012)  J Asthma  PELICAN | Chronic Lower Respiratory Diseases | Title PELICAN: a cluster-randomized controlled trial in Dutch general practices to assess a self-management support intervention based on individual goals for children with asthma  Location/Funding UK/Europe Non-govt/non-industry  Setting Clinic  Intervention Type Individualized self management  Unit of Analysis Patient  N Clusters 9 | Patient N enrolled: 33  Mean Age: Arm 1: 8.4 years; Arm 2: 8.7 years  Sex % female: Arm 1: 33.3%; Arm 2: 42.9%  Race/Ethnicity: Arm 1: African – 6.7%; White – 93.3%  Arm 2: White – 100%; African – 0%  Socioeconomic Status:  Arm 1:  Low 0%; Middle 13.3%; High 86.7%  Arm 2:  Low 7.1%; Middle 28.6%; High 64.3% | Primary Outcomes:  PRO HRQL of the child (measured by the self-administered Paediatric Asthma-related Quality of Life Questionnaire – standardized; PAQLQ-s |
| Verbene, 2015(Verbene, 2015 #830)  Eur J Surg Oncol | Cancer | Title Intensified follow-up in colorectal cancer patients using frequent Carcino-Embryonic Antigen (CEA) measurements and CEA-triggered imaging: Results of the randomized "CEAwatch" trial  Location/Funding UK/Europe Government  Setting Hospital  Intervention Type Quality improvement  Unit of Analysis Patient  N Clusters 11 | Patient N enrolled: 3,223  Median Age at Diagnosis: 70 years  Sex Total % female: 44%  Race/Ethnicity: Unclear  Socioeconomic Status: Unclear | Primary Outcomes:  Clinical The number of recurrences per follow-up arm, the proportion of recurrences that could be treated with curative intent, the proportion of recurrences with definitive curative treatment outcome (R0 resection of all recurrent disease), and the time to detection of recurrent disease. |
| Wagner, 2014(Wagner, 2014 #967)  J Clin Oncol | Cancer | Title Nurse navigators in early cancer care: a randomized, controlled trial  Location/Funding U.S. Government  Setting Clinic  Intervention Type Nurse Navigators  Unit of Analysis Provider  N Clusters 109 | Patient N enrolled: 251  Mean Age: Arm 1: 64.4 years; Arm 2: 60.4 years  Sex % female: Arm 1: 86.4%; Arm 2: 91%  Race/Ethnicity:  Arm 1: African-American – 2.5%; American Indian – 4.2%; Asian – 4.2%; Hawaiian – 0.9%; White – 84.8%; Other – 2.5%  Arm 2: African-American – 5.3%; American Indian – 5.3%; Asian – 7.5%; Hawaiian – 0%; White – 79.7%; Other – 2.3%  Socioeconomic Status:  Education  Arm 1:  < High school - 2.5%; High school graduate or GED - 19.5%; Some college - 33.9%; College graduate - 20.3%; Postgraduate degree - 23.7% Arm 2:  < High school - 1.5%; High school graduate or GED - 9.8%; Some college - 25.8%; College graduate - 42.4%; Postgraduate degree - 20.5% | Primary Outcomes:  Primary outcome not clearly stated. |
| Walters, 2013(Walters, 2013 #508)  BMJ Open | Chronic Lower Respiratory Diseases | Title Effects of telephone health mentoring in community-recruited chronic obstructive pulmonary disease on self-management capacity, quality of life and psychological morbidity: a randomised controlled trial  Location/Funding Australia/N.Z. Government, Non-govt/non-industry  Setting Clinic  Intervention Type Behavioral, Quality improvement  Unit of Analysis Clinic  N Clusters 31 | Patient N enrolled: 182  Mean Age: Arm 1: 67.3 years; Arm 2: 68.2 years  Sex % female: Arm 1: 49%; Arm 2: 46%  Race/Ethnicity: Unclear  Socioeconomic Status:  Education N  Arm 1:  Less than or = to 10: 69  Year 11-12: 7  Postschool Qualification: 16  Currently Employed: 17 Arm 2: Less than or = to 10: 60  Year 11-12: 12  Postschool Qualification: 18  Currently Employed: 17 | Primary Outcomes:  PRO Health Related Quality of Life |
| Wang, 2015(Wang, 2015 #186)  Circ Cardiovasc Interv  TRANSLATE-POPS | Cardiovascular Disease | Title Cluster-randomized clinical trial examining the impact of platelet function testing on practice: the treatment with adenosine diphosphate receptor inhibitors: longitudinal assessment of treatment patterns and events after acute coronary syndrome prospective open label antiplatelet therapy study  Location/Funding U.S. Industry  Setting Hospital  Intervention Type Quality improvement  Unit of Analysis Patient  N Clusters 100 | Patient N enrolled: 3,817  Median Age:  Arm 1: 59 years;  Arm 2: 60 years  Sex % female:  Arm 1: 28.9%;  Arm 2: 25.4%  Race/Ethnicity:  Arm 1: White – 88.7%;  Arm 2: White – 87.3%  Socioeconomic Status: Unclear | Primary Outcomes:  Clinical The frequency of ADPri therapy adjustment during the index MI hospitalization. |
| Williams, 2010(Williams, 2010 #202)  J Allergy Clin Immunol | Chronic Lower Respiratory Diseases | Title A cluster-randomized trial to provide clinicians inhaled corticosteroid adherence information for their patients with asthma  Location/Funding U.S. Government, Non-govt/non-industry  Setting Clinic  Intervention Type Behavioral  Unit of Analysis Patient  N Clusters 34 | Patient N enrolled: 2,698  Median Age:  Arm 1: 5-17years - 44.3%; 18-56 - 55.7%  Arm 2: 5-17years - 44.3%; 18-56 - 55.7%  Sex % female:  Arm 1: 55.2%;  Arm 2: 55.3%  Race/Ethnicity:  Arm 1: African-American – 38.3%; White – 54.4%; Other - 7.3%  Arm 2: African-American – 38.7%; White – 55%; Other – 6.3%  Socioeconomic Status: Unclear | Primary Outcomes:  Behavioral Patient adherence to ICSs in the last 3 months of intervention |
| Zimmermann, 2014(Zimmermann, 2014 #368)  Lancet | Cancer | Title Early palliative care for patients with advanced cancer: a cluster-randomised controlled trial  Location/Funding Canada Government, Non-govt/non-industry  Setting Clinic  Intervention Type Behavioral, Quality improvement  Unit of Analysis Patient  N Clusters 24 | Patient N enrolled: 461  Mean Age:  Arm 1: 61.2 years  Arm 2: 60.2 years  Sex % female: Arm 1: 59.6%; Arm 2: 53.6%  Race/Ethnicity: Unclear  Socioeconomic Status:  Education  Arm 1:  Below high school – 8.8%  High school – 24.8%  College, university, or other – 67.3%  Arm 2:  Below high school – 10.3%  High school – 24.6%  College, university, or other – 65.1% | Primary Outcomes:  PRO Quality of life measured by functional assessment of chronic illness therapy-spiritual well being [FACIT-Sp]  Change from baseline to 3 months was the primary outcome |
| Zwar, 2012(Zwar, 2012 #108)  Med J Aust | Chronic Lower Respiratory Diseases | Title Care of patients with a diagnosis of chronic obstructive pulmonary disease: a cluster randomised controlled trial  Location/Funding Australia/N.Z. Unclear/NR  Setting Clinic  Intervention Type Quality improvement  Unit of Analysis Patient  N Clusters 44 | Patient N enrolled: 451  Mean Age: Arm 1: 65.8 years; Arm 2: 64.4 years  Sex % female: Arm 1: 53%; Arm 2: 51.2%  Race/Ethnicity: Unclear  Socioeconomic Status: Unclear | Primary Outcomes:  PRO Disease related quality of life (St. George's Respiratory Questionnaire [SGRQ]) |

Abbreviations: HRQL=health-related quality of life, NR=not reported, PRO=patient-reported outcome,
