# Supplementary material for: Assessing heterogeneity of treatment effect analyses in health-related cluster randomized trials: A systematic review
Source: PLoS One. 2019 Aug 12;14(8):e0219894. doi: 10.1371/journal.pone.0219894 (PMC6690528; doi:10.1371/journal.pone.0219894)
Supplement: S4 Table — (DOCX) [file pone.0219894.s005.docx]

**S4 Table: ICC and Power Estimate for Included CRTs**

| **Statistical Design** | **All** | **Cancer** | **Cardiovascular** | **Pulmonary** |
| --- | --- | --- | --- | --- |
|  | N= 64 | N= 16 | N= 18 | N=30 |
| # patients analyzed (median, IQR) | 447 25% IQR: 234.25 75% IQR: 1134 | 264.4 25% IQR: 202.5 75% IQR: 516.5 | 1405 25% IQR: 637 75% IQR: 3537.75 | 397.5 25% IQR: 172.5 75% IQR: 798.5 |
| # clusters analyzed (median, IQR) | 40  25% IQR: 17 75% IQR: 90 | 17  25% IQR: 12 75% IQR: 87.75 | 88  25% IQR: 39 75% IQR: 168 | 37 25% IQR: 21.5 75% IQR: 49.25 |
| Power, mean, SD | 81.99% (SD: 4.4%) | 80% (SD: 0) | 84.25% (SD: 6.3%) | 81.3% (SD: 3.3%) |
| Alpha | 43 (67.1%) | 9 (56.25%) | 13 (72.2%) | 21 (70%) |
| one-sided | 2 | 1 | 0 | 1 |
| two-sided | 17 | 3 | 10 | 4 |
| not listed | 24 | 5 | 3 | 16 |
| ICC presented | 35 (54.6%) | 7 (43.75%) | 10 (55.5%) | 18 (60%) |
| Percent presented | 1%-28.3% | 1% - 5% | 1%-28.3% | 1%-10% |
| mean ICC, SD, range | 0.05 (0.01 - 0.283) | 0.04 (0.01 - 0.05) | 0.09 (0.01 - 0.283) | 0.04 (0.01 - 0.1) |
