# Supplementary material for: Assessing heterogeneity of treatment effect analyses in health-related cluster randomized trials: A systematic review
Source: PLoS One. 2019 Aug 12;14(8):e0219894. doi: 10.1371/journal.pone.0219894 (PMC6690528; doi:10.1371/journal.pone.0219894)
Supplement: S5 Table — (DOCX) [file pone.0219894.s006.docx]

**S5 Table: Design and Reporting Quality for Included Health Systems CRTs**

| **Quality Assessment and Reporting Quality** | **All** | **Cancer** | **Cardiovascular** | **Pulmonary** |
| --- | --- | --- | --- | --- |
| **Quality Assessment** | N =64 | N = 16 | N = 18 | N = 30 |
| Justification for cluster design, n (%) | 24 (37.5%) | 8 (50%) | 5 (27.7%) | 11 (36.6%) |
| Uses at least 4 clusters per treatment group | 57 (89%) | 13 (81.25%) | 16 (88.9%) | 28 (93.3%) |
| Allows for clustering in sample size | 39 (60%) | 10 (62.5%) | 12 (66.7%) | 17 (54.8%) |
| Uses matching, stratification, or minimization | 37 (57.8%) | 6 (37.5%) | 12 (66.6%) | 19 (63.3%) |
| Allows of clustering in analysis | 49 (76.5%) | 14 (87.5%) | 14 (77.8%) | 21 (70%) |
| **Reporting Quality** |  |  |  |  |
| Cluster RCT in title | 36 (56.25%) | 11 (68.75%) | 8 (44.4%) | 17 (56.6%) |
| ICC estimate included | 39 (60.9%) | 10 (62.5%) | 10 (55.6%) | 19 (63.3%) |
| Lists number of clusters randomized | 60 (93.75%) | 15 (93.75%) | 17 (94.4%) | 28 (93.3%) |
| Describes baseline comparison of clusters | 20 (31.25%) | 5 (31.25%) | 5 (27.8%) | 10 (33.3%) |
| Describes baseline comparison of individuals | 62 (95.4%) | 16 (100%) | 18 (100%) | 28 (93.3%) |
| Average cluster size listed | 23 (35.9%) | 6 (37.5%) | 6 (33.3%) | 11 (36.6%) |
| Explains whether analysis conducted at the cluster or individual level | 38 (59.3%) | 10 (62.5%) | 8 (44.4%) | 20 (66.6%) |
| Reports on loss to follow up of clusters | 56 (87.5%) | 13 (81.25%) | 17 (94.4%) | 26 (86.6%) |
| Reports on loss to follow up individuals | 59 (92.1%) | 15 (93.75%) | 17 (94.4%) | 27 (90%) |
| *100% Reporting Quality* | *5 (7.8%)* | *1 (6.25%)* | *1 (5.5%)* | *3 (10%)* |
