# Supplementary material for: Assessing heterogeneity of treatment effect analyses in health-related cluster randomized trials: A systematic review
Source: PLoS One. 2019 Aug 12;14(8):e0219894. doi: 10.1371/journal.pone.0219894 (PMC6690528; doi:10.1371/journal.pone.0219894)
Supplement: S1 Text — (DOCX) [file pone.0219894.s009.docx]

S1 Text. List of Included Studies

Abernethy AP, Currow DC, Shelby-James T, et al. Delivery strategies to optimize resource utilization and performance status for patients with advanced life-limiting illness: results from the "palliative care trial" [ISRCTN 81117481]. J Pain Symptom Manage 2013;45(3):488-505. DOI: 10.1016/j.jpainsymman.2012.02.024. PMID: 23102711.

Abramson MJ, Schattner RL, Sulaiman ND, et al. Do spirometry and regular follow-up improve health outcomes in general practice patients with asthma or COPD? A cluster randomised controlled trial. Med J Aust 2010;193(2):104-9. PMID: 20642418.

Adams J, Cymbala AA, Delate T, et al. Cluster-Randomized Trial of Clinical Pharmacist Tobacco Cessation Counseling Among Patients with Cardiovascular Disease. Popul Health Manag 2015;18(4):300-6. DOI: 10.1089/pop.2014.0106. PMID: 25647441.

Al-sheyab N, Gallagher R, Crisp J, et al. Peer-led education for adolescents with asthma in Jordan: a cluster-randomized controlled trial. Pediatrics 2012;129(1):e106-12. DOI: 10.1542/peds.2011-0346. PMID: 22157137.

Armour CL, Reddel HK, LeMay KS, et al. Feasibility and effectiveness of an evidence-based asthma service in Australian community pharmacies: a pragmatic cluster randomized trial. J Asthma 2013;50(3):302-9. DOI: 10.3109/02770903.2012.754463. PMID: 23270495.

Au DH, Udris EM, Engelberg RA, et al. A randomized trial to improve communication about end-of-life care among patients with COPD. Chest 2012;141(3):726-35. DOI: 10.1378/chest.11-0362. PMID: 21940765.

Austin MA, Wills KE, Blizzard L, et al. Effect of high flow oxygen on mortality in chronic obstructive pulmonary disease patients in prehospital setting: randomised controlled trial. Bmj 2010;341:c5462. DOI: 10.1136/bmj.c5462. PMID: 20959284.

Bell LM, Grundmeier R, Localio R, et al. Electronic health record-based decision support to improve asthma care: a cluster-randomized trial. Pediatrics 2010;125(4):e770-7. DOI: 10.1542/peds.2009-1385. PMID: 20231191.

Benger J, Coates D, Davies S, et al. Randomised comparison of the effectiveness of the laryngeal mask airway supreme, i-gel and current practice in the initial airway management of out of hospital cardiac arrest: a feasibility study. Br J Anaesth 2016;116(2):262-8. DOI: 10.1093/bja/aev477. PMID: 26787796.

Bergholdt SH, Larsen PV, Kragstrup J, et al. Enhanced involvement of general practitioners in cancer rehabilitation: a randomised controlled trial. BMJ Open 2012;2(2):e000764. DOI: 10.1136/bmjopen-2011-000764. PMID: 22508956.

Berwanger O, Guimaraes HP, Laranjeira LN, et al. Effect of a multifaceted intervention on use of evidence-based therapies in patients with acute coronary syndromes in Brazil: the BRIDGE-ACS randomized trial. Jama 2012;307(19):2041-9. DOI: 10.1001/jama.2012.413. PMID: 22665103.

Brotons C, Soriano N, Moral I, et al. Randomized clinical trial to assess the efficacy of a comprehensive programme of secondary prevention of cardiovascular disease in general practice: the PREseAP study. Rev Esp Cardiol 2011;64(1):13-20. DOI: 10.1016/j.recesp.2010.07.005. PMID: 21194823.

Casey D, Murphy K, Devane D, et al. The effectiveness of a structured education pulmonary rehabilitation programme for improving the health status of people with moderate and severe chronic obstructive pulmonary disease in primary care: the PRINCE cluster randomised trial. Thorax 2013;68(10):922-8. DOI: 10.1136/thoraxjnl-2012-203103. PMID: 23736156.

Costantini M, Romoli V, Leo SD, et al. Liverpool Care Pathway for patients with cancer in hospital: a cluster randomised trial. Lancet 2014;383(9913):226-37. DOI: 10.1016/s0140-6736(13)61725-0. PMID: 24139708.

Dear RF, Barratt AL, Askie LM, et al. Impact of a cancer clinical trials web site on discussions about trial participation: a cluster randomized trial. Ann Oncol 2012;23(7):1912-8. DOI: 10.1093/annonc/mdr585. PMID: 22258366.

Du X, Gao R, Turnbull F, et al. Hospital quality improvement initiative for patients with acute coronary syndromes in China: a cluster randomized, controlled trial. Circ Cardiovasc Qual Outcomes 2014;7(2):217-26. DOI: 10.1161/circoutcomes.113.000526. PMID: 24619325.

Fihn SD, Bucher JB, McDonell M, et al. Collaborative care intervention for stable ischemic heart disease. Arch Intern Med 2011;171(16):1471-9. DOI: 10.1001/archinternmed.2011.372. PMID: 21911632.

Flather MD, Babalis D, Booth J, et al. Cluster-randomized trial to evaluate the effects of a quality improvement program on management of non-ST-elevation acute coronary syndromes: The European Quality Improvement Programme for Acute Coronary Syndromes (EQUIP-ACS). Am Heart J 2011;162(4):700-707.e1. DOI: 10.1016/j.ahj.2011.07.027. PMID: 21982663.

Foster JM, Usherwood T, Smith L, et al. Inhaler reminders improve adherence with controller treatment in primary care patients with asthma. J Allergy Clin Immunol 2014;134(6):1260-1268.e3. DOI: 10.1016/j.jaci.2014.05.041. PMID: 25062783.

Garbutt JM, Yan Y, Highstein G, et al. A cluster-randomized trial shows telephone peer coaching for parents reduces children's asthma morbidity. J Allergy Clin Immunol 2015;135(5):1163-70.e1-2. DOI: 10.1016/j.jaci.2014.09.033. PMID: 25445827.

Garcia-Cardenas V, Sabater-Hernandez D, Kenny P, et al. Effect of a pharmacist intervention on asthma control. A cluster randomised trial. Respir Med 2013;107(9):1346-55. DOI: 10.1016/j.rmed.2013.05.014. PMID: 23810267.

Guldbrandt LM, Fenger-Gron M, Rasmussen TR, et al. The effect of direct access to CT scan in early lung cancer detection: an unblinded, cluster-randomised trial. BMC Cancer 2015;15:934. DOI: 10.1186/s12885-015-1941-2. PMID: 26608727.

Hilberink SR, Jacobs JE, Breteler MH, et al. General practice counseling for patients with chronic obstructive pulmonary disease to quit smoking: impact after 1 year of two complex interventions. Patient Educ Couns 2011;83(1):120-4. DOI: 10.1016/j.pec.2010.04.009. PMID: 20430565.

Holton C, Crockett A, Nelson M, et al. Does spirometry training in general practice improve quality and outcomes of asthma care?. Int J Qual Health Care 2011;23(5):545-53. DOI: 10.1093/intqhc/mzr039. PMID: 21733979.

Holton CH, Beilby JJ, Harris MF, et al. Systematic care for asthma in Australian general practice: a randomised controlled trial. Med J Aust 2010;193(6):332-7. PMID: 20854237.

Honkoop PJ, Loijmans RJ, Termeer EH, et al. Symptom- and fraction of exhaled nitric oxide-driven strategies for asthma control: A cluster-randomized trial in primary care. J Allergy Clin Immunol 2015;135(3):682-8.e11. DOI: 10.1111/dme.12574; 10.1016/j.jaci.2014.07.016. PMID: 25174865.

Hopkinson JB, Fenlon DR, Okamoto I, et al. The deliverability, acceptability, and perceived effect of the Macmillan approach to weight loss and eating difficulties: a phase II, cluster-randomized, exploratory trial of a psychosocial intervention for weight- and eating-related distress in people with advanced cancer. J Pain Symptom Manage 2010;40(5):684-95. DOI: 10.1016/j.jpainsymman.2010.02.015. PMID: 20678895.

Hostler D, Everson-Stewart S, Rea TD, et al. Effect of real-time feedback during cardiopulmonary resuscitation outside hospital: prospective, cluster-randomised trial. Bmj 2011;342: d512. DOI: 10.1136/bmj.d512. PMID: 21296838.

Jahn P, Kuss O, Schmidt H, et al. Improvement of pain-related self-management for cancer patients through a modular transitional nursing intervention: a cluster-randomized multicenter trial. Pain 2014;155(4):746-54. DOI: 10.1016/j.pain.2014.01.006. PMID: 24434732.

Janson SL, McGrath KW, Covington JK, et al. Objective airway monitoring improves asthma control in the cold and flu season: a cluster randomized trial. Chest 2010;138(5):1148-55. DOI: 10.1378/chest.09-2394. PMID: 20538819.

Jiang YQ, Zhu YX, Chen XL, et al. Impact of adherence to GOLD guidelines on 6-minute walk distance, MRC dyspnea scale score, lung function decline, quality of life, and quality-adjusted life years in a Shanghai suburb. Genet Mol Res 2015;14(3):8861-70. DOI: 10.4238/2015.August.3.9. PMID: 26345817.

Kinsman LD, Rotter T, Willis J, et al. Do clinical pathways enhance access to evidence-based acute myocardial infarction treatment in rural emergency departments?. Aust J Rural Health 2012;20(2):59-66. DOI: 10.1111/j.1440-1584.2012.01262.x. PMID: 22435765.

Kruis AL, Boland MR, Assendelft WJ, et al. Effectiveness of integrated disease management for primary care chronic obstructive pulmonary disease patients: results of cluster randomised trial. Bmj 2014;349:g5392. DOI: 10.1177/1460458214546773; 10.1136/bmj.g5392. PMID: 25209620.

Krum H, Forbes A, Yallop J, et al. Telephone support to rural and remote patients with heart failure: the Chronic Heart Failure Assessment by Telephone (CHAT) study. Cardiovasc Ther 2013;31(4):230-7. DOI: 10.1111/1755-5922.12009. PMID: 23061492.

Levine DA, Funkhouser EM, Houston TK, et al. Improving care after myocardial infarction using a 2-year internet-delivered intervention: the Department of Veterans Affairs myocardial infarction-plus cluster-randomized trial. Arch Intern Med 2011;171(21):1910-7. DOI: 10.1001/archinternmed.2011.498. PMID: 22123798.

Livingston PM, Craike MJ, Salmon J, et al. Effects of a clinician referral and exercise program for men who have completed active treatment for prostate cancer: A multicenter cluster randomized controlled trial (ENGAGE). Cancer 2015;121(15):2646-54. DOI: 10.1002/cncr.29385. PMID: 25877784.

Lowrie R, Mair FS, Greenlaw N, et al. Pharmacist intervention in primary care to improve outcomes in patients with left ventricular systolic dysfunction. Eur Heart J 2012;33(3):314-24. DOI: 10.1093/eurheartj/ehr433. PMID: 22083873.

McCorkle R, Jeon S, Ercolano E, et al. An Advanced Practice Nurse Coordinated Multidisciplinary Intervention for Patients with Late-Stage Cancer: A Cluster Randomized Trial. J Palliat Med 2015;18(11):962-9. DOI: 10.1089/jpm.2015.0113. PMID: 26305992.

Meng K, Musekamp G, Schuler M, et al. The impact of a self-management patient education program for patients with chronic heart failure undergoing inpatient cardiac rehabilitation. Patient Educ Couns 2016. DOI: 10.1016/j.pec.2016.02.010. PMID: 26898600.

Nichol G, Leroux B, Wang H, et al. Trial of Continuous or Interrupted Chest Compressions during CPR. N Engl J Med 2015;373(23):2203-14. DOI: 10.1056/NEJMoa1509139. PMID: 26550795.

Nicolaije KA, Ezendam NP, Vos MC, et al. Impact of an Automatically Generated Cancer Survivorship Care Plan on Patient-Reported Outcomes in Routine Clinical Practice: Longitudinal Outcomes of a Pragmatic, Cluster Randomized Trial. J Clin Oncol 2015;33(31):3550-9. DOI: 10.1200/jco.2014.60.3399. PMID: 26304900.

Nokela M, Arnlind MH, Ehrs PO, et al. The influence of structured information and monitoring on the outcome of asthma treatment in primary care: a cluster randomized study. Respiration 2010;79(5):388-94. DOI: 10.1159/000235548. PMID: 19672055.

Ono Y, Hayakawa M, Maekawa K, et al. Should laryngeal tubes or masks be used for out-of-hospital cardiac arrest patients?. Am J Emerg Med 2015;33(10):1360-3. DOI: 10.1016/j.ajem.2015.07.043. PMID: 26306437.

Overbeek LI, Hermens RP, van Krieken JH, et al. Electronic reminders for pathologists promote recognition of patients at risk for Lynch syndrome: cluster-randomised controlled trial. Virchows Arch 2010;456(6):653-9. DOI: 10.1007/s00428-010-0907-7. PMID: 20379742.

Perkins GD, Lall R, Quinn T, et al. Mechanical versus manual chest compression for out-of-hospital cardiac arrest (PARAMEDIC): a pragmatic, cluster randomised controlled trial. Lancet 2015;385(9972):947-55. DOI: 10.1016/s0140-6736(14)61886-9. PMID: 25467566.

Rixon L, Hirani SP, Cartwright M, et al. A RCT of telehealth for COPD patient's quality of life: the whole system demonstrator evaluation. Clin Respir J 2015. DOI: 10.1111/crj.12359. PMID: 26260325.

Schwalm JD, Ivers NM, Natarajan MK, et al. Cluster randomized controlled trial of Delayed Educational Reminders for Long-term Medication Adherence in ST-Elevation Myocardial Infarction (DERLA-STEMI). Am Heart J 2015;170(5):903-13. DOI: 10.1016/j.ahj.2015.08.014. PMID: 26542498.

Simunovic M, Coates A, Goldsmith CH, et al. The cluster-randomized Quality Initiative in Rectal Cancer trial: evaluating a quality-improvement strategy in surgery. Cmaj 2010;182(12):1301-6. DOI: 10.1503/cmaj.091883. PMID: 20696797.

Smidth M, Christensen MB, Fenger-Gron M, et al. The effect of an active implementation of a disease management programme for chronic obstructive pulmonary disease on healthcare utilization--a cluster-randomised controlled trial. BMC Health Serv Res 2013;13:385. DOI: 10.1186/1472-6963-13-385. PMID: 24090189.

Smidth M, Olesen F, Fenger-Gron M, et al. Patient-experienced effect of an active implementation of a disease management programme for COPD - a randomised trial. BMC Fam Pract 2013;14:147. DOI: 10.1186/1471-2296-14-147. PMID: 24088417.

Smith JR, Noble MJ, Musgrave S, et al. The at-risk registers in severe asthma (ARRISA) study: a cluster-randomised controlled trial examining effectiveness and costs in primary care. Thorax 2012;67(12):1052-60. DOI: 10.1136/thoraxjnl-2012-202093. PMID: 22941976.

Stiell IG, Nichol G, Leroux BG, et al. Early versus later rhythm analysis in patients with out-of-hospital cardiac arrest. N Engl J Med 2011;365(9):787-97. DOI: 10.1056/NEJMoa1010076. PMID: 21879896.

Stout JW, Smith K, Zhou C, et al. Learning from a distance: effectiveness of online spirometry training in improving asthma care. Acad Pediatr 2012;12(2):88-95. DOI: 10.1016/j.acap.2011.11.006. PMID: 22424397.

Strasser F, Blum D, von Moos R, et al. The effect of real-time electronic monitoring of patient-reported symptoms and clinical syndromes in outpatient workflow of medical oncologists: E-MOSAIC, a multicenter cluster-randomized phase III study (SAKK 95/06). Ann Oncol 2016;27(2):324-32. DOI: 10.1093/annonc/mdv576. PMID: 26646758.

Sulaiman ND, Barton CA, Liaw ST, et al. Do small group workshops and locally adapted guidelines improve asthma patients' health outcomes? A cluster randomized controlled trial. Fam Pract 2010;27(3):246-54. DOI: 10.1093/fampra/cmq013. PMID: 20332178.

Tamblyn R, Ernst P, Winslade N, et al. Evaluating the impact of an integrated computer-based decision support with person-centered analytics for the management of asthma in primary care: a randomized controlled trial. J Am Med Inform Assoc 2015;22(4):773-83. DOI: 10.1093/jamia/ocu009. PMID: 25670755.

van Bragt S, van den Bemt L, Kievits R, et al. PELICAN: a cluster-randomized controlled trial in Dutch general practices to assess a self-management support intervention based on individual goals for children with asthma. J Asthma 2015;52(2):211-9. DOI: 10.1186/1745-6215-15-341; 10.3109/02770903.2014.952439. PMID: 25166455.

Verberne CJ, Zhan Z, van den Heuvel E, et al. Intensified follow-up in colorectal cancer patients using frequent Carcino-Embryonic Antigen (CEA) measurements and CEA-triggered imaging: Results of the randomized "CEAwatch" trial. Eur J Surg Oncol 2015;41(9):1188-96. DOI: 10.1161/circulationaha.115.015373; 10.1016/j.ejso.2015.06.008. PMID: 26184850.

Wagner EH, Ludman EJ, Aiello Bowles EJ, et al. Nurse navigators in early cancer care: a randomized, controlled trial. J Clin Oncol 2014;32(1):12-8. DOI: 10.1200/jco.2013.51.7359. PMID: 24276777.

Walters J, Cameron-Tucker H, Wills K, et al. Effects of telephone health mentoring in community-recruited chronic obstructive pulmonary disease on self-management capacity, quality of life and psychological morbidity: a randomised controlled trial. BMJ Open 2013;3(9):e003097. DOI: 10.1136/bmjopen-2013-003097. PMID: 24014482.

Wang TY, Henry TD, Effron MB, et al. Cluster-randomized clinical trial examining the impact of platelet function testing on practice: the treatment with adenosine diphosphate receptor inhibitors: longitudinal assessment of treatment patterns and events after acute coronary syndrome prospective open label antiplatelet therapy study. Circ Cardiovasc Interv 2015;8(6):e001712. DOI: 10.1161/circinterventions.114.001712. PMID: 26025216.

Williams LK, Peterson EL, Wells K, et al. A cluster-randomized trial to provide clinicians inhaled corticosteroid adherence information for their patients with asthma. J Allergy Clin Immunol 2010;126(2):225-31, 231.e1-4. DOI: 10.1016/j.jaci.2010.03.034. PMID: 20569973.

Zimmermann C, Swami N, Krzyzanowska M, et al. Early palliative care for patients with advanced cancer: a cluster-randomised controlled trial. Lancet 2014;383(9930):1721-30. DOI: 10.1016/s0140-6736(13)62416-2. PMID: 24559581.

Zwar NA, Hermiz O, Comino E, et al. Care of patients with a diagnosis of chronic obstructive pulmonary disease: a cluster randomised controlled trial. Med J Aust 2012;197(7):394-8. PMID: 23025736.
