# Supplementary material for: Assessing heterogeneity of treatment effect analyses in health-related cluster randomized trials: A systematic review
Source: PLoS One. 2019 Aug 12;14(8):e0219894. doi: 10.1371/journal.pone.0219894 (PMC6690528; doi:10.1371/journal.pone.0219894)
Supplement: S2 Text — (DOCX) [file pone.0219894.s010.docx]

**S2 Text. List of Excluded Studies**

All studies listed below were reviewed in their full-text version and excluded for the reasons cited. Reasons for exclusion signify only the usefulness of the articles for this study and are not intended as criticisms of the articles.

**Not a publication type of interest**

Boxer RS, Dolansky MA, Bodnar CA, et al. A randomized trial of heart failure disease management in skilled nursing facilities: design and rationale. J Am Med Dir Assoc 2013;14(9):710.e5 -11. DOI: 10.1016/j.jamda.2013.05.023. PMID: 23871475.

Costantini M, Ottonelli S, Canavacci L, et al. The effectiveness of the Liverpool care pathway in improving end of life care for dying cancer patients in hospital. A cluster randomised trial. BMC Health Serv Res 2011;11:13. DOI: 10.1186/1472-6963-11-13. PMID: 21261949.

De Vera MA, Sadatsafavi M, Tsao NW, et al. Empowering pharmacists in asthma management through interactive SMS (EmPhAsIS): study protocol for a randomized controlled trial. Trials 2014;15:488. DOI: 10.1186/1745-6215-15-488. PMID: 25494702.

Greer A and Tully RP. Mechanical versus manual chest compression for out-of-hospital cardiac arrest (PARAMEDIC): A pragmatic, cluster randomised control trial. Journal of the Intensive Care Society 2015;16(3):241-243.

Houben CH, Spruit MA, Wouters EF, et al. A randomised controlled trial on the efficacy of advance care planning on the quality of end-of-life care and communication in patients with COPD: the research protocol. BMJ Open 2014;4(1):e004465. DOI: 10.1136/bmjopen-2013-004465. PMID: 24384905.

Kintner E, Cook G, Marti CN, et al. Comparative Effectiveness on Cognitive Asthma Outcomes of the SHARP Academic Asthma Health Education and Counseling Program and a Non-Academic Program. Res Nurs Health 2015;38(6):423-35. DOI: 10.1002/nur.21678. PMID: 26296595.

Kintner EK, Cook G, Marti CN, et al. Effectiveness of a school-based academic asthma health education and counseling program on fostering acceptance of asthma in older school-age students with asthma. J Spec Pediatr Nurs 2015;20(1):49-61. DOI: 10.1111/jspn.12098. PMID: 25443593.

Manfrin A, Thomas T and Krska J. Randomised evaluation of the Italian medicines use review provided by community pharmacists using asthma as a model (RE I-MUR). BMC Health Serv Res 2015;15:171. DOI: 10.1186/s12913-015-0791-6. PMID: 25896873.

Myers MG, Godwin M, Dawes M, et al. Conventional versus automated measurement of blood pressure in the office (CAMBO) trial. Fam Pract 2012;29(4):376-82. DOI: 10.1093/fampra/cmr113. PMID: 22117083.

Steventon A, Bardsley M, Billings J, et al. Effect of telehealth on use of secondary care and mortality: findings from the Whole System Demonstrator cluster randomised trial. Bmj 2012;344:e3874. DOI: 10.1136/bmj.e3874. PMID: 22723612.

Valk MJ, Hoes AW, Mosterd A, et al. Rationale, design and baseline results of the Treatment Optimisation in Primary care of Heart failure in the Utrecht region (TOPHU) study: a cluster randomised controlled trial. BMC Fam Pract 2015;16(1):130. DOI: 10.1186/s12875-015-0347-1. PMID: 26446696.

**Not a cluster-randomized trial**

Jabre P, Tazarourte K, Azoulay E, et al. Offering the opportunity for family to be present during cardiopulmonary resuscitation: 1-year assessment. Intensive Care Med 2014;40(7):981-7. DOI: 10.1007/s00134-014-3337-1. PMID: 24852952.

Ram S, Narayanasamy R and Barua A. Effectiveness of Group Psycho-education on Well-being and Depression Among Breast Cancer Survivors of Melaka, Malaysia. Indian J Palliat Care 2013;19(1):34-9. DOI: 10.4103/0973-1075.110234. PMID: 23766593.

Russell IT, Edwards RT, Gliddon AE, et al. Cancer of Oesophagus or Gastricus - New Assessment of Technology of Endosonography (COGNATE): report of pragmatic randomised trial. Health Technol Assess 2013;17(39):1-170. DOI: 10.3310/hta17390. PMID: 24034150.

**Study population is not individuals with a disease of interest**

Beune EJ, Moll van Charante EP, Beem L, et al. Culturally adapted hypertension education (CAHE) to improve blood pressure control and treatment adherence in patients of African origin with uncontrolled hypertension: cluster-randomized trial. PLoS One 2014;9(3):e90103. DOI: 10.1371/journal.pone.0090103. PMID: 24598584.

Black DA, Taggart J, Jayasinghe UW, et al. The Teamwork Study: enhancing the role of non-GP staff in chronic disease management in general practice. Aust J Prim Health 2013;19(3):184-9. DOI: 10.1071/py11071. PMID: 22951281.

Carter BL, Coffey CS, Ardery G, et al. Cluster-randomized trial of a physician/pharmacist collaborative model to improve blood pressure control. Circ Cardiovasc Qual Outcomes 2015;8(3):235-43. DOI: 10.1161/circoutcomes.114.001283. PMID: 25805647.

Carter BL, Doucette WR, Franciscus CL, et al. Deterioration of blood pressure control after discontinuation of a physician-pharmacist collaborative intervention. Pharmacotherapy 2010;30(3):228-35. DOI: 10.1592/phco.30.3.228. PMID: 20180606.

Freund T, Peters-Klimm F, Boyd CM, et al. Medical Assistant-Based Care Management for High-Risk Patients in Small Primary Care Practices: A Cluster Randomized Clinical Trial. Ann Intern Med 2016;164(5):323-30. DOI: 10.7326/m14-2403. PMID: 26833209.

Gonzales R, Anderer T, McCulloch CE, et al. A cluster randomized trial of decision support strategies for reducing antibiotic use in acute bronchitis. JAMA Intern Med 2013;173(4):267-73. DOI: 10.1001/jamainternmed.2013.1589. PMID: 23319069.

Griffin SJ, Borch-Johnsen K, Davies MJ, et al. Effect of early intensive multifactorial therapy on 5-year cardiovascular outcomes in individuals with type 2 diabetes detected by screening (ADDITION-Europe): a cluster-randomised trial. Lancet 2011;378(9786):156-67. DOI: 10.1016/s0140-6736(11)60698-3. PMID: 21705063.

Guldbrandt LM. The effect of direct referral for fast CT scan in early lung cancer detection in general practice. A clinical, cluster-randomised trial. Dan Med J 2015;62(3). PMID: 25748876.

Inadomi JM, Vijan S, Janz NK, et al. Adherence to colorectal cancer screening: a randomized clinical trial of competing strategies. Arch Intern Med 2012;172(7):575-82. DOI: 10.1001/archinternmed.2012.332. PMID: 22493463.

Jafar TH, Islam M, Hatcher J, et al. Community based lifestyle intervention for blood pressure reduction in children and young adults in developing country: cluster randomised controlled trial. Bmj 2010;340:c2641. DOI: 10.1136/bmj.c2641. PMID: 20530082.

Keller H, Krones T, Becker A, et al. Arriba: effects of an educational intervention on prescribing behaviour in prevention of CVD in general practice. Eur J Prev Cardiol 2012;19(3):322-9. DOI: 10.1177/1741826711404502. PMID: 21450565.

Labhardt ND, Balo JR, Ndam M, et al. Improved retention rates with low-cost interventions in hypertension and diabetes management in a rural African environment of nurse-led care: a cluster-randomised trial. Trop Med Int Health 2011;16(10):1276-84. DOI: 10.1111/j.1365-3156.2011.02827.x. PMID: 21733046.

LaBresh KA, Ariza AJ, Lazorick S, et al. Adoption of cardiovascular risk reduction guidelines: a cluster-randomized trial. Pediatrics 2014;134(3):e732-8. DOI: 10.3109/02770903.2014.952439; 10.1542/peds.2014-0876. PMID: 25157013.

Li G, Zhang P, Wang J, et al. Cardiovascular mortality, all-cause mortality, and diabetes incidence after lifestyle intervention for people with impaired glucose tolerance in the Da Qing Diabetes Prevention Study: a 23-year follow-up study. Lancet Diabetes Endocrinol 2014;2(6):474-80. DOI: 10.1016/s2213-8587(14)70057-9. PMID: 24731674.

Lusignan S, Gallagher H, Jones S, et al. Audit-based education lowers systolic blood pressure in chronic kidney disease: the Quality Improvement in CKD (QICKD) trial results. Kidney Int 2013;84(3):609-20. DOI: 10.1038/ki.2013.96. PMID: 23536132.

Margolis KL, Asche SE, Bergdall AR, et al. Effect of home blood pressure telemonitoring and pharmacist management on blood pressure control: a cluster randomized clinical trial. Jama 2013;310(1):46-56. DOI: 10.1001/jama.2013.6549. PMID: 23821088.

Morgan MA, Coates MJ, Dunbar JA, et al. The TrueBlue model of collaborative care using practice nurses as case managers for depression alongside diabetes or heart disease: a randomised trial. BMJ Open 2013;3(1). DOI: 10.1136/bmjopen-2012-002171. PMID: 23355671.

Ogedegbe G, Tobin JN, Fernandez S, et al. Counseling African Americans to Control Hypertension: cluster-randomized clinical trial main effects. Circulation 2014;129(20):2044-51. DOI: 10.1161/circulationaha.113.006650. PMID: 24657991.

Patja K, Absetz P, Auvinen A, et al. Health coaching by telephony to support self-care in chronic diseases: clinical outcomes from The TERVA randomized controlled trial. BMC Health Serv Res 2012;12:147. DOI: 10.1186/1472-6963-12-147. PMID: 22682298.

Tomonaga Y, Gutzwiller F, Luscher TF, et al. Diagnostic accuracy of point-of-care testing for acute coronary syndromes, heart failure and thromboembolic events in primary care: a cluster-randomised controlled trial. BMC Fam Pract 2011;12:12. DOI: 10.1186/1471-2296-12-12. PMID: 21435203.

van der Weegen S and Verwey R. It's LiFe! Mobile and Web-Based Monitoring and Feedback Tool Embedded in Primary Care Increases Physical Activity: A Cluster Randomized Controlled Trial. 2015;17(7):e184. DOI: 10.1186/s12889-015-2059-9; 10.2196/jmir.4579. PMID: 26209025.

Weidman EK, Bell G, Walsh D, et al. Assessing the impact of immersive simulation on clinical performance during actual in-hospital cardiac arrest with CPR-sensing technology: A randomized feasibility study. Resuscitation 2010;81(11):1556-61. DOI: 10.1016/j.resuscitation.2010.05.021. PMID: 20724057.

Wells KJ, Lee JH, Calcano ER, et al. A cluster randomized trial evaluating the efficacy of patient navigation in improving quality of diagnostic care for patients with breast or colorectal cancer abnormalities. Cancer Epidemiol Biomarkers Prev 2012;21(10):1664-72. DOI: 10.1158/1055-9965.epi-12-0448. PMID: 23045541.

Zhou Y, Hu G, Wang D, et al. Community based integrated intervention for prevention and management of chronic obstructive pulmonary disease (COPD) in Guangdong, China: cluster randomised controlled trial. Bmj 2010;341:c6387. DOI: 10.1136/bmj.c6387. PMID: 21123342.

**Does not report any patient-level outcomes**

DeVore AD, Cox M, Heidenreich PA, et al. Cluster-Randomized Trial of Personalized Site Performance Feedback in Get With The Guidelines-Heart Failure. Circ Cardiovasc Qual Outcomes 2015;8(4):421-7. DOI: 10.1161/circoutcomes.114.001333. PMID: 26175533.

Ezendam NP, Nicolaije KA, Kruitwagen RF, et al. Survivorship Care Plans to inform the primary care physician: results from the ROGY care pragmatic cluster randomized controlled trial. J Cancer Surviv 2014;8(4):595-602. DOI: 10.1007/s11764-014-0368-0. PMID: 24866923.

Kintner EK, Cook G, Marti CN, et al. Effectiveness of a school- and community-based academic asthma health education program on use of effective asthma self-care behaviors in older school-age students. J Spec Pediatr Nurs 2015;20(1):62-75. DOI: 10.1111/jspn.12099. PMID: 25443867.

Mold JW, Fox C, Wisniewski A, et al. Implementing asthma guidelines using practice facilitation and local learning collaboratives: a randomized controlled trial. Ann Fam Med 2014;12(3):233-40. DOI: 10.1370/afm.1624. PMID: 24821894.

Newhouse RP, Dennison Himmelfarb C, Morlock L, et al. A phased cluster-randomized trial of rural hospitals testing a quality collaborative to improve heart failure care: organizational context matters. Med Care 2013;51(5):396-403. DOI: 10.1097/MLR.0b013e318286e32e. PMID: 23579349.

Weiser C, van Tulder R, Stockl M, et al. Dispatchers impression plus Medical Priority Dispatch System reduced dispatch centre times in cases of out of hospital cardiac arrest. Pre-alert--a prospective, cluster randomized trial. Resuscitation 2013;84(7):883-8. DOI: 10.1016/j.resuscitation.2012.12.017. PMID: 23295777.

**Not the primary/main report of the study results**

Abramson MJ, Schattner RL, Holton C, et al. Spirometry and regular follow-up do not improve quality of life in children or adolescents with asthma: Cluster randomized controlled trials. Pediatr Pulmonol 2015;50(10):947-54. DOI: 10.1002/ppul.23096. PMID: 25200397.

Bergholdt SH, Hansen DG, Larsen PV, et al. A randomised controlled trial to improve the role of the general practitioner in cancer rehabilitation: effect on patients' satisfaction with their general practitioners. BMJ Open 2013;3(7). DOI: 10.1136/bmjopen-2013-002726. PMID: 23824312.

Bergholdt SH, Sondergaard J, Larsen PV, et al. A randomised controlled trial to improve general practitioners' services in cancer rehabilitation: effects on general practitioners' proactivity and on patients' participation in rehabilitation activities. Acta Oncol 2013;52(2):400-9. DOI: 10.3109/0284186x.2012.741711. PMID: 23173759.

Cartwright M, Hirani SP, Rixon L, et al. Effect of telehealth on quality of life and psychological outcomes over 12 months (Whole Systems Demonstrator telehealth questionnaire study): nested study of patient reported outcomes in a pragmatic, cluster randomised controlled trial. Bmj 2013;346:f653. DOI: 10.1136/bmj.f653. PMID: 23444424.

Craike M, Gaskin CJ, Courneya KS, et al. Predictors of adherence to a 12-week exercise program among men treated for prostate cancer: ENGAGE study. Cancer Med 2016. DOI: 10.1002/cam4.639. PMID: 26872005.

Foster JM, Smith L, Usherwood T, et al. General practitioner-delivered adherence counseling in asthma: feasibility and usefulness of skills, training and support tools. J Asthma 2016;53(3):311-20. DOI: 10.3109/02770903.2015.1091473. PMID: 26365203.

Murphy AW, Cupples ME, Murphy E, et al. Six-year follow-up of the SPHERE RCT: secondary prevention of heart disease in general practice. BMJ Open 2015;5(11):e007807. DOI: 10.1136/bmjopen-2015-007807. PMID: 26534729.

Wadhwa D, Burman D, Swami N, et al. Quality of life and mental health in caregivers of outpatients with advanced cancer. Psychooncology 2013;22(2):403-10. DOI: 10.1002/pon.2104. PMID: 22135229.

Wentlandt K, Burman D, Swami N, et al. Preparation for the end of life in patients with advanced cancer and association with communication with professional caregivers. Psychooncology 2012;21(8):868-76. DOI: 10.1002/pon.1995. PMID: 21648015.
